# Coupling nitrate capture with ammonia production through bifunctional redox-electrodes

**DOI:** 10.1038/s41467-023-36318-1

**Published:** 2023-02-14

**Authors:** Kwiyong Kim, Alexandra Zagalskaya, Jing Lian Ng, Jaeyoung Hong, Vitaly Alexandrov, Tuan Anh Pham, Xiao Su

**Affiliations:** 1 Department of Chemical and Biomolecular Engineering, University of Illinois Urbana-Champaign, Urbana, IL 61801 USA; 2 Quantum Simulations Group, Materials Science Division, Lawrence Livermore National Laboratory, Livermore, CA 94550 USA; 3 Department of Chemical and Biomolecular Engineering, University of Nebraska-Lincoln, Lincoln, NE 68588 USA; 4 Department of Materials Science and Engineering, University of Illinois at Urbana-Champaign, Urbana, IL 61801 USA; 5 Nebraska Center for Materials and Nanoscience, University of Nebraska-Lincoln, Lincoln, NE 68588 USA; 6 Laboratory for Energy Applications for the Future (LEAF), Lawrence Livermore National Laboratory, Livermore, CA 94550 USA; 7 Present Address: Department of Urban and Environmental Engineering, Graduate School of Carbon Neutrality, Ulsan National Institute of Science and Technology (UNIST), 50 UNIST-gil, Eonyang-eup, Ulju-gun, Ulsan, 44919 Republic of Korea

**Keywords:** Energy science and technology, Pollution remediation, Electrocatalysis, Materials for energy and catalysis

## Abstract

Nitrate is a ubiquitous aqueous pollutant from agricultural and industrial activities. At the same time, conversion of nitrate to ammonia provides an attractive solution for the coupled environmental and energy challenge underlying the nitrogen cycle, by valorizing a pollutant to a carbon-free energy carrier and essential chemical feedstock. Mass transport limitations are a key obstacle to the efficient conversion of nitrate to ammonia from water streams, due to the dilute concentration of nitrate. Here, we develop bifunctional electrodes that couple a nitrate-selective redox-electrosorbent (polyaniline) with an electrocatalyst (cobalt oxide) for nitrate to ammonium conversion. We demonstrate the synergistic reactive separation of nitrate through solely electrochemical control. Electrochemically-reversible nitrate uptake greater than 70 mg/g can be achieved, with electronic structure calculations and spectroscopic measurements providing insight into the underlying role of hydrogen bonding for nitrate selectivity. Using agricultural tile drainage water containing dilute nitrate (0.27 mM), we demonstrate that the bifunctional electrode can achieve a 8-fold up-concentration of nitrate, a 24-fold enhancement of ammonium production rate (108.1 ug h^−1^ cm^−2^), and a >10-fold enhancement in energy efficiency when compared to direct electrocatalysis in the dilute stream. Our study provides a generalized strategy for a fully electrified reaction-separation pathway for modular nitrate remediation and ammonia production.

## Abstract (web summary)

Coupling nitrate capture with conversion to ammonium is attractive because it combines pollutant remediation and chemical production. Here, the authors design a bifunctional redox-electrode to achieve the reactive separation of nitrate to ammonium in a single electrochemical cell.

## Introduction

Nitrate, because of its mobility, water solubility, and persistence, has long been recognized as a widespread macropollutant that leads to eutrophication and algal blooms^1–3^. Nitrate pollution from drinking water in the United States has been identified as a major cause of cancer, so a maximum contaminant level (MCL) of 11.3 and 10 mg/L NO_3_-N is recommended by the World Health Organization (WHO) and US Environmental Protection Agency (EPA), respectively^3,4^. Nitrate pollution is mainly caused by the overuse of reactive nitrogen-based fertilizers and nitrogen runoffs from agricultural use^3,5,6^. Nitrogen loss by leaching and runoff is estimated to reach 132 Tg N yr^−1^ in 2030^7^, and the transport of nitrate from rivers to oceans accounts for 40–70 Tg N yr^−1^^8^. Several water purification methods have been proposed for the removal of nitrate, including ion exchange or reverse osmosis^9^, but these methods can often be limited by high energy consumption, waste generation, and limitations in selectivity and capacity.

**Fig. 1:**
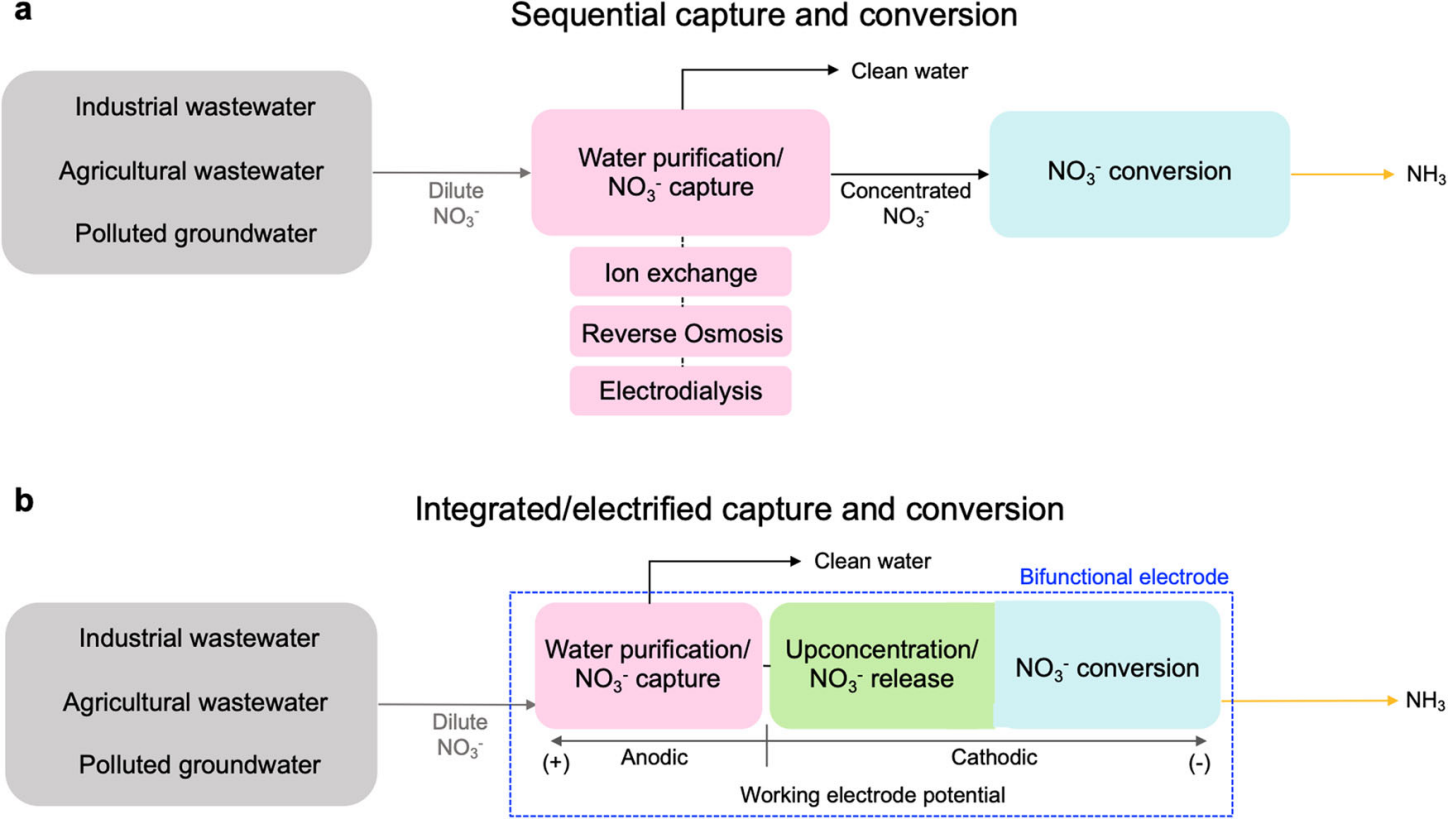
Sequential and integrated pathway for dilute nitrate capture and conversion. **a** A schematic illustration of the sequential route of physicochemical purification (ion exchange, reverse osmosis, and electrodialysis) and electrochemical conversion. **b** A schematic illustration of integrated nitrate capture, up-concentration, and conversion using a composite of a redox-active electrosorbent and a metal oxide electrocatalyst proposed in this study.

At the same time, nitrate can be a promising nitrogen source for ammonia production, in which a pollutant can be valorized into an energy carrier and fertilizer^10,11^. In comparison with the triple N≡N bond in dinitrogen (941 kJ mol^−1^), the N=O bond has a lower dissociation energy (204 kJ mol^−1^), leading to faster ammonia production kinetics compared to dinitrogen^11–14^. In this context, electrochemical conversion of nitrate to ammonia has been proposed as a decentralized and sustainable alternative to the energy- and carbon-intensive Haber–Bosch process, which contributes to 2% of the world’s energy consumption, and 1–2% of the carbon dioxide emissions^15–19^. Recent years have seen notable developments in enabling electrochemical nitrate reduction, but most of the electrochemical studies have used model or synthetic nitrate concentrations in a higher range (10–1000 mM) to evaluate the performance of catalysts and devices (Supplementary Table 1). However, in natural environments or even wastewater, nitrate concentrations are usually much more dilute, thus presenting significant transport barriers for (electro)catalytic performance irrespective of catalyst nature. While some point sources, such as nuclear waste, may contain relatively high levels of nitrate, the availability of nitrate-rich waste streams is limited^10^, and such sources may contain interfering metal species, complicating the conversion step^20^. In most cases, nitrate streams, such as those resulting from industrial or agricultural runoff or polluted groundwater, contain much lower concentrations of nitrate than being currently used in the electrochemical studies (Supplementary Table 1). Within the United States, typical nitrate levels in agricultural groundwater range between 0.2 and 0.4 mM, and about 80% of agricultural wells have nitrate concentrations less than the MCL of 10 mg/L NO_3_-N (0.714 mM) set by EPA^4^. Unfortunately, electrocatalysis can run into a significant extent of side reactions (e.g., hydrogen evolution), particularly under such low nitrate concentrations^21^, which thus severely affecting the energy efficiency. Furthermore, the low ionic conductivity of dilute nitrate waste sources requires the integration of an additional separation step, to remove and concentrate the nitrate before efficient electroconversion can occur (Fig. 1a)^10^.

**Fig. 2:**
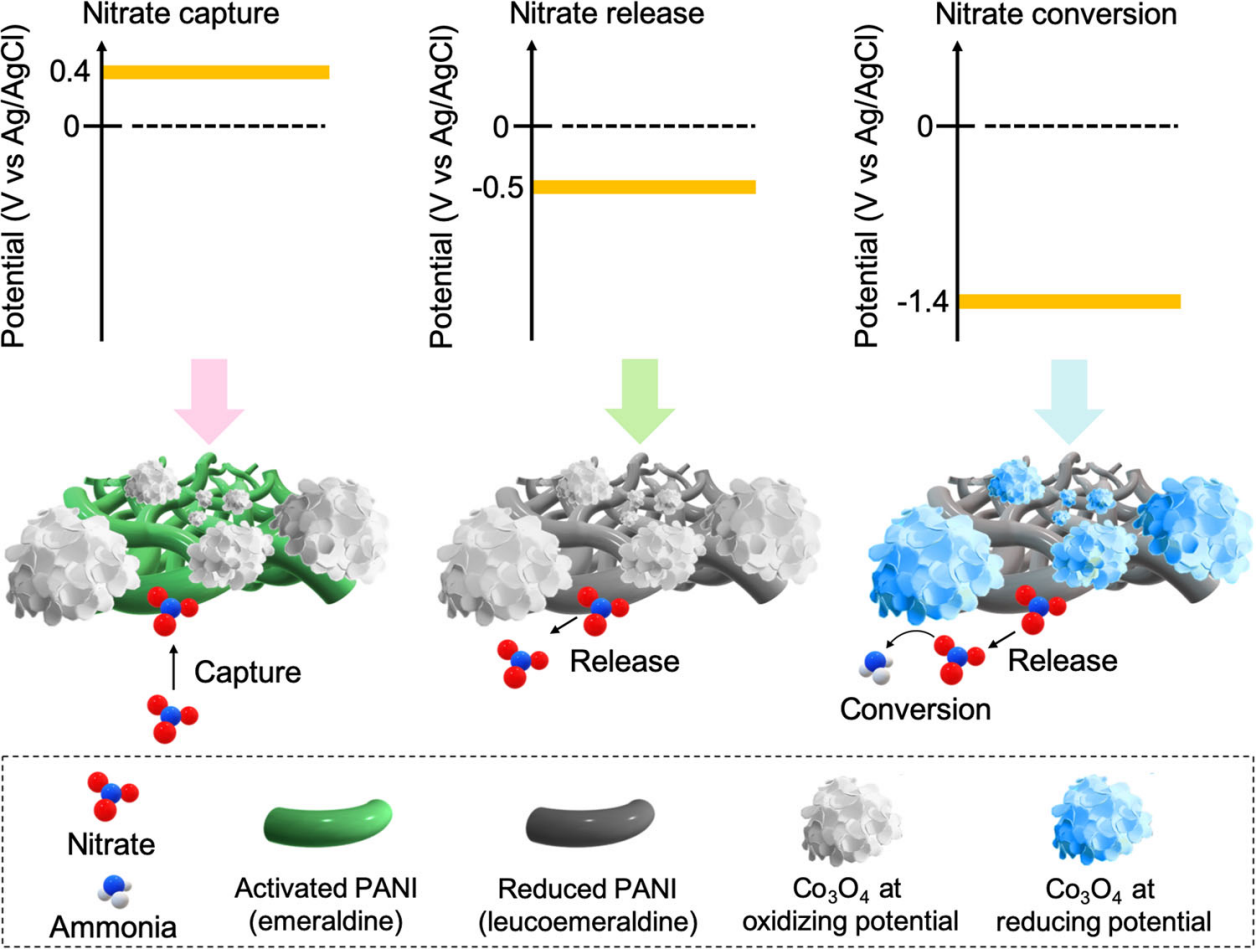
Schematic illustration of bifunctional PANI-Co_3_O_4_/CNT electrode. Upon anodic charging at +0.4 V vs Ag/AgCl, PANI is activated (emeraldine) and servs as an electrosorbent for nitrate. During cathodic charging, PANI is reduced to leucoemeraldine form, and adsorbed nitrate is released (−0.5 V vs Ag/AgCl), or PANI regeneration is coupled with nitrate electroconversion to ammonia by Co_3_O_4_ (−1.4 V vs Ag/AgCl).

To overcome these intrinsic challenges, we couple nitrate separation and reaction through a bifunctional electrode, to enable nitrogen valorization within a single electrochemical cell. We report an all-electrified approach for the synergistic coupling of dilute nitrate capture, up-concentration and conversion to ammonia (Fig. 1b), to enhance the energy efficiency and reduce the capital cost of valorizing dilute nitrate streams. We combine a redox-active polymer (polyaniline, PANI) with a metal oxide catalyst (cobalt oxide, Co_3_O_4_) supported on carbon nanotube (CNT), to serve as a nanostructured, bifunctional electrosorbent and electrocatalyst (Fig. 2). The composite redox-electrode allows the integration of separation, regeneration/up-concentration, and electrocatalysis in a single electrochemical device under isothermal conditions, without the need to separately generate and transport concentrated nitrate, and equally importantly, with no use of chemical regeneration during the separation step.

Electrosorption based on redox-active materials has proven to be effective for ion-selective separations by combining molecularly-controllable affinity, modularity, scalability, and no need for chemical reagents for regeneraton^22–27^. While these materials have been recently used for various contaminant remediation strategies^22–24,28^, we explore for the first time the applicability of these redox processes for the electrified capture and downstream conversion of nitrogen species. To impart electrocatalytic properties into our electrode, we selected cobalt oxide (Co_3_O_4_) as a model nitrate-reducing electrocatalyst based on its activity and durability under various pH-potential conditions^29–32^. Furthermore, during our synthesis strategy, we took advantage of cobalt oxide’s intrinsic low electrical conductivity to spatially control the electropolymerization of PANI on conductive CNT, without covering the catalytic surface of Co_3_O_4_. Thus, to achieve a true bifunctional electrode design, both PANI and Co_3_O_4_ were exposed to the electrolyte (Fig. 2), and the separation (by PANI) and reaction (by Co_3_O_4_) performances did not negatively interfere with each other. Here, we utilize CO_3_O_4_ as a robust and facile platform for proof-of-concept in the current work—however, the concept is generalizable and other nitrate electroreduction electrocatalysts could be leveraged in future work.

Within this work, first we demonstrated that polyaniline (PANI) enabled the selective capture of nitrate via ion-exchange and hydrogen bonding in a synergistic manner. Through a combination of electrosorption measurement, electronic structure calculations, and spectroscopy, we pursued a mechanistic investigation to elucidate the structural influence of different PANI species (leucoemeraldine, emeraldine, and pernigranline) on nitrate selectivity over competing species. Next, by electrochemically releasing the bound nitrate, our composite electrodes (PANI-Co_3_O_4_/CNT) generated a localized nitrate-rich receiving stream with suitable conductivity for electroreduction, thus enabling electrocatalytic conversion of nitrate to ammonium (half-cell reaction: NO_3_^−^ + 6H_2_O + 8e^−^ → NH_3_ + 9OH^−^) with significantly lower energy consumption, and enhanced faradaic efficiency. Furthermore, the electrosorbent regeneration could be coupled directly with nitrate reduction by solely controlling release potential (Fig. 2), enabling process intensification in a modular fashion. Through a single plug-and-play electrochemical reactor enabling reactive separation, this study shows how tailored bifunctional redox electrodes can achieve process intensification for nitrogen valorization. Also, our concept fundamentally explores how integrating separation can overcome transport limitations from low nitrate concentrations, with a proof-of-concept demonstration directly using agricultural water matrices with natural occurring nitrate concentration. By combining electro-driven separation with electrocatalysis, we can enable process intensification through solely electrochemical pathways, paving the possible way for a fully decentralized, renewable-driven nitrate remediation and ammonium production system.

## Results

### Characterization of PANI-Co_3_O_4_/CNT composites

**Fig. 3:**
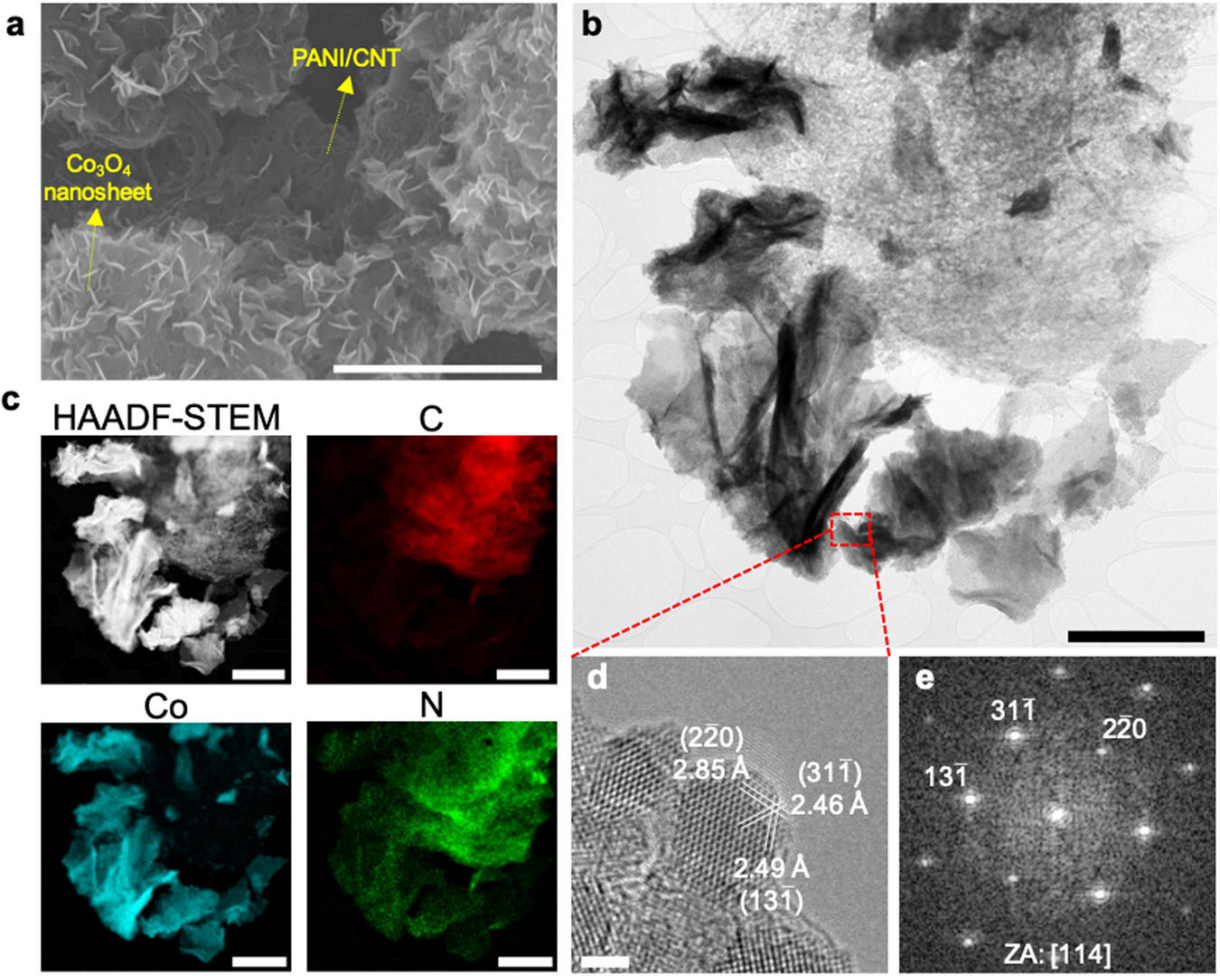
Morphology characterization of PANI-CO_3_O_4_/CNT. **a** A scanning electron microscopy (SEM) image of PANI-Co_3_O_4_/CNT. **b** A transmission electron microscopy (TEM) and **c** high-angle annular dark-field scanning transmission electron microscopy (HAADF-STEM) image and corresponding energy-dispersive spectroscopy (EDS) mapping images of PANI-Co_3_O_4_/CNT. Cobalt nanosheets and PANI/CNT fibers exist in separate domains, making two materials exposed to an electrolyte. **d** High-resolution TEM (HRTEM) image and **e** corresponding fast Fourier transform (FFT) of a Co_3_O_4_ particle aligned at [114] zone axis (ZA). Plane indices are denoted on both (**d**) and (**e**) along with corresponding interplanar distances in (**d**). Scale bars: 3 μm for (**a**), 1 μm for (**b**, **c**), and 2 nm for (**d**).

A heterostructure of PANI-Co_3_O_4_/CNT was synthesized by first electrodepositing Co(OH)_2_ on CNT, followed by heat treatment and subsequent PANI electropolymerization (Supplementary Fig. 1). As depicted in Supplementary Fig. 2, X-ray diffraction (XRD) analysis confirmed that Co(OH)_2_ was obtained by electrodeposition in 0.1 M Co(NO_3_)_2_ at −1.0 V vs Ag/AgCl (ICSD No. 88940), which was then converted to Co_3_O_4_ by heat treatment (ICSD No. 36256). We observed that even after anodic electropolymerization of PANI under acidic conditions, the XRD pattern of PANI-Co_3_O_4_/CNTs was not changed, indicating that the crystal structure of Co_3_O_4_ was maintained during the coating of PANI. Transmission electron microscopy (TEM) and scanning electron microscopy (SEM) were used to investigate the morphology of PANI/CNT and PANI-Co_3_O_4_/CNT composites. For PANI/CNT, a thin amorphous PANI layer was formed onto cylindrical CNT during the electropolymerization (Supplementary Figs. 3–6). The TEM and SEM images of PANI-Co_3_O_4_/CNT showed that the composite was constructed from distinct domains of Co_3_O_4_ nanosheets, which were spatially separated from one another (Fig. 3a, b and Supplementary Fig. 7). As Co(OH)_2_ is electrodeposited, the existing particles may serve as active spots on which successive deposition takes place preferentially, thus resulting in locally grown clusters of nanosheets. On the other hand, because PANI electropolymerizes only on conductive surfaces and Co_3_O_4_ is not electrically conductive^33^, PANI was not coated on Co_3_O_4_ but rather on adjacent CNT. Energy-dispersive X-ray spectroscopy (EDS) analysis (Fig. 3c) confirmed that PANI-Co_3_O_4_/CNT composites contained both PANI/CNT layers and Co_3_O_4_ with nanosheets morphology in distinct domains. According to the electron energy loss spectroscopy (EELS) analysis, a clear nitrogen peak at 402 eV appeared on the surface of the PANI/CNT in the PANI-Co_3_O_4_/CNT, which was not detectable on the surface of the Co_3_O_4_ nanosheet (Supplementary Fig. 8). With clear accessibility to the electrolyte solution, the bifunctional electrode facilitates their dual function as an electrosorbent and electrocatalyst. High-resolution TEM (HRTEM) analysis in Fig. 3d, e confirms that the nanosheets are comprised of Co_3_O_4_ nanoparticles.

### Selective electrosorption of nitrate by polyaniline-electrodes

**Fig. 4:**
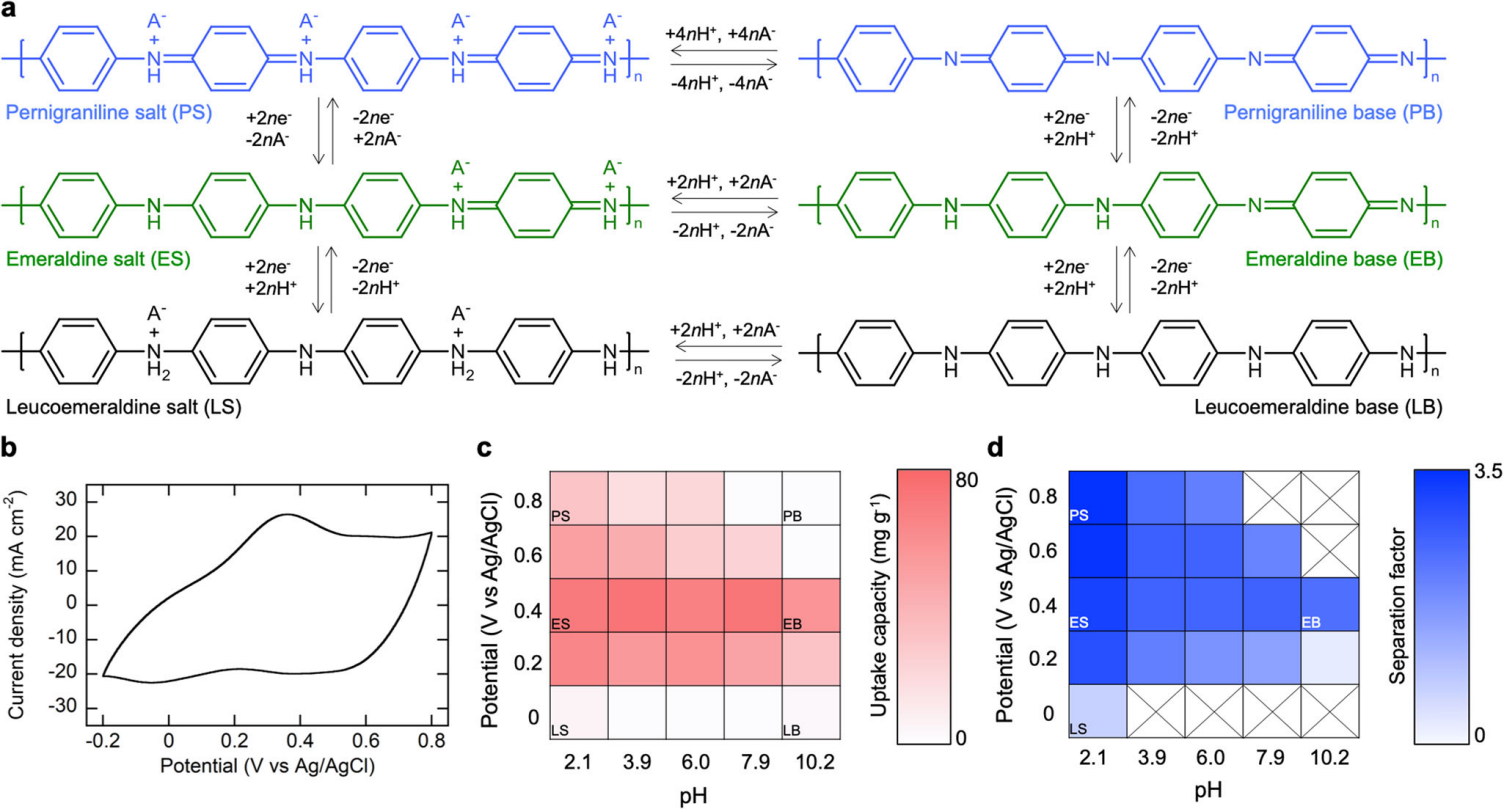
Structural effect of PANI species (leucoemeraldine, emeraldine, and pernigraniline) on selective capture of nitrate. **a** Redox-interconversion of PANI caused by the change in electrode potential and solution pH. **b** A cyclic voltammogram of a PANI/CNT electrode in 0.5 M H_2_SO_4_. Scan rate was 5 mV s^−1^. **c** A heatmap of nitrate uptake capacity (mg NO_3_^−^ g^−1^ PANI) at various pH and electrode potential. Both NO_3_^−^ and Cl^−^ concentrations were 5 mM. **d** A heatmap of separation factor (SF) of PANI toward nitrate over chloride at various pH and electrode potential. Both NO_3_^−^ and Cl^−^ concentrations were 5 mM. LS leucoemeraldine salt, LB leucoemeraldine base, ES emeraldine salt, EB emeraldine base, PS pernigraniline salt, PB pernigraniline base.

Despite prior studies exploring the use of PANI as an adsorbent for nitrate^34–36^, there has been little mechanistic understanding of how PANI speciation affects selectivity and uptake. Figure 4a shows the redox-interconversion of PANI with different oxidation states (leucoemeraldine, emeraldine, and pernigraniline) and their respective protonation. As shown in a cyclic voltammogram (CV) of PANI/CNT in 0.5 M H_2_SO_4_ (Fig. 4b), the first major anodic peak at +0.3 V vs Ag/AgCl can be attributed to the oxidation of leucoemeraldine to emeraldine, and another oxidation onset at about +0.73 V vs Ag/AgCl can be assigned to the oxidation of emeraldine to pernigraniline^37^. Depending on the pH of the solution, each oxidation state can be protonated into “salt” or deprotonated into “base”, thus generating six different forms (LS, LB, ES, EB, PS, and PB) (Fig. 4a) of PANI^38^.

The affinity of six different forms of PANI to nitrate was examined by using PANI/CNT as heterogeneous electrosorbents, in solutions containing 5 mM NO_3_^−^ and equimolar Cl^−^ as a competing species. By varying pH and electrochemical potential for electrosorption, a heatmap was constructed for nitrate uptake (mg NO_3_^−^ g^−1^ PANI) (Fig. 4c). Regardless of pH, nitrate uptake was negligible at 0 V vs Ag/AgCl, since reducing PANI to leucoemeraldine generated negative current, and the resulting electrostatic repulsion impedes the electrostatic approach of nitrate to the working electrode. In order to obtain a better understanding of the heatmap, the point of zero charge (PZC) of PANI/CNTs with different oxidation states was determined by using a pH drift method^34^. As shown in Supplementary Fig. 9, the observed PZCs of PANI films polarized at +0.4 and +0.8 V vs Ag/AgCl (as a representative of emeraldine and pernigraniline, respectively) were 4.20 and 3.60, respectively. PZC analysis indicated that pernigraniline PANI (+0.8 V vs Ag/AgCl) carried a positive charge only at pH <3.60, possibly from protonated quinoid imine groups (-NH^+^=), which may act as an ion-exchange site for nitrate uptake (Fig. 4a). As equilibrium pH increased, deprotonation occurred during the conversion of −NH^+^= to −N=, resulting in the loss of ion-exchange sites. Compared to pernigraniline, we observed that emeraldine has a slightly broader pH window where PANI exists in protonated salt (Supplementary Fig. 9). Interestingly, at a higher pH range (>7), nitrate binding still occurred on emeraldine at +0.4 V vs Ag/AgCl (Fig. 4c). At these pH and potential levels, PANI was expected to exist primarily as EB, which does not possess a positively charged −NH^+^= groups. We therefore attributed the uptake to hydrogen bonding via benzoid amine (−NH−) in EB. Conversely, at high pH (>7), pernigraniline (+0.8 V vs Ag/AgCl) has only quinoid imines (−N=), which does not contain hydrogen bond sites, so nitrate adsorption did not take place at high pH and high oxidation potentials (Fig. 4c)—as will be elucidated below by DFT calculations. The ES form, especially at +0.4 V vs Ag/AgCl, provided the best capacity for nitrate uptake (>70 mg NO_3_^−1^ g^−1^ PANI) through synergistic ion-exchange and hydrogen bonding. X-ray photoelectron spectroscopy (XPS) survey scan showed a signal for S2*p* in a pristine PANI/CNT, which indicates the presence of sulfate (SO_4_^2−^) as a counter ion doped during the PANI electropolymerization step in sulfuric acid (Supplementary Fig. 10). Compared to the pristine electrode (N:S:Cl = 75.6:24.4:0), PANI/CNT after electrosorption exhibited a decreased atomic ratio of sulfur (N:S:Cl = 85.1:4.7:10.2), yet an increase in nitrogen and chloride, demonstrating that ion-exchange occurred. Attenuated total reflection infrared (ATR-IR) spectroscopy analysis (Supplementary Fig. 11) revealed that emeraldine PANI treated in 1 M NaOH for 24 h —regarded as EB—exhibited a shoulder of N–H stretch at 3388 cm^−1^, which is a characteristic of the free (not hydrogen-bonded) N–H groups when PANI is in its undoped state. Due to intermolecular hydrogen bonding^39^, EB also exhibits a peak of hydrogen-bonded N–H groups at about 3300 cm^−1^. On the other hand, in emeraldine PANI treated in 1 M NaNO_3_ for 24 h, there was shift to lower wavenumber (3225 cm^−1^), which corresponds to the stretching vibration of the secondary amine N–H group hydrogen bonded with nitrate, in alignment with the previous observations from the literature on H-bonding^40,41^.

We also constructed a heatmap for separation factors (SFs) obtained using PANI/CNT at various combinations of pH and potential to see the structural dependence of selectivity between nitrate and chloride (Fig. 4d, see “Methods” for calculation details of SF). Some combinations which resulted in negligible uptake (<5 mg g^−1^) were excluded. In general, lower pH resulted in higher SF toward nitrate compared to chloride, reaching a SF of 3.45 at PS (0.8 V vs Ag/AgCl, pH = 2.1). In terms of oxidation state, the more oxidized salt (higher potential) exhibited better selectivity toward nitrate, and LS exhibited higher affinity toward chloride (SF: 0.79 at 0 V vs Ag/AgCl, pH = 2.1) (Fig. 4d).

### Mechanistic study for nitrate affinity and selectivity on PANI-electrodes

**Fig. 5:**
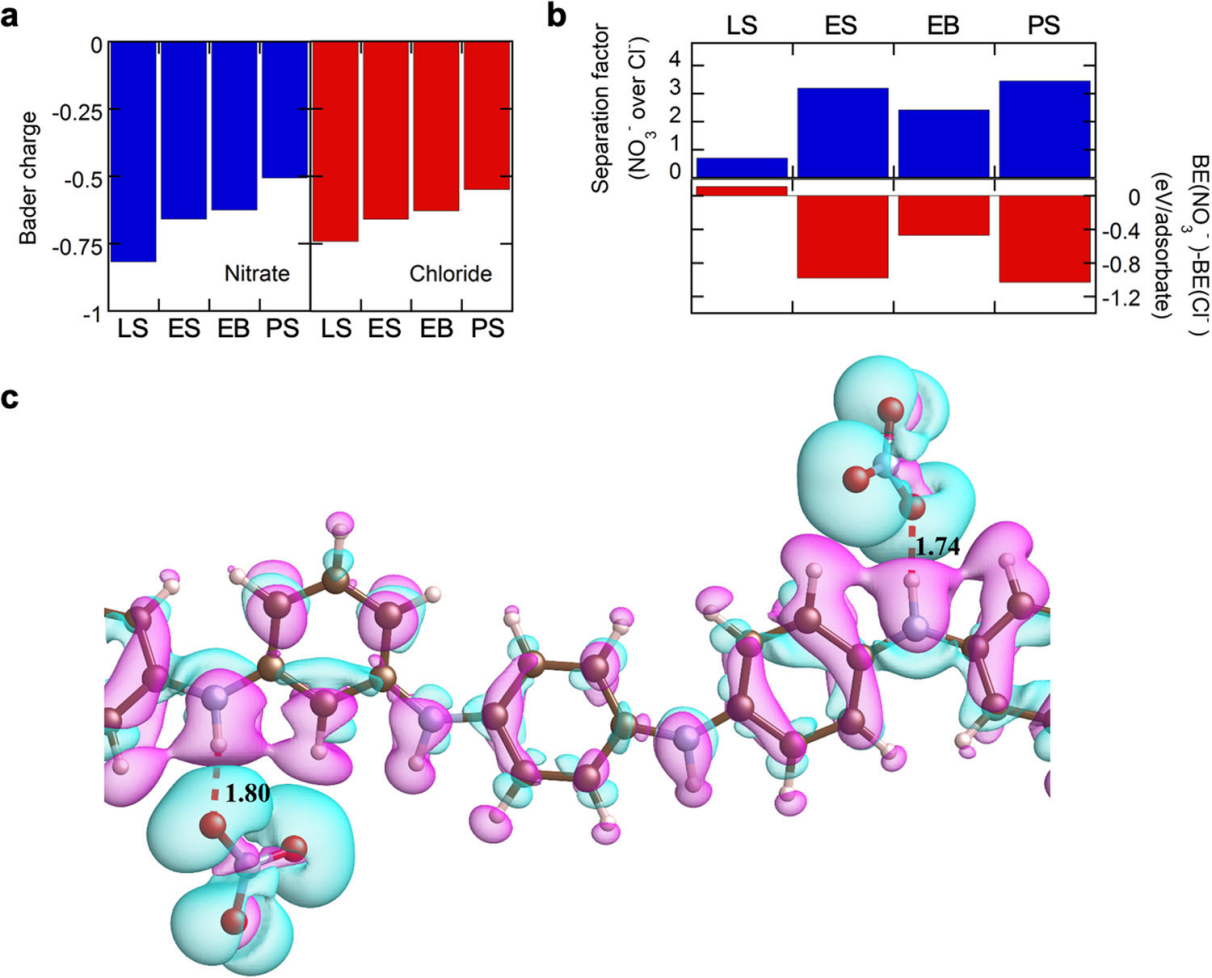
Electronic structure calculations on the binding between PANI and nitrate. **a** Atomic Bader charges of NO_3_^−^/Cl^−^ species bonded to the PANI. The total Bader charge was calculated on the optimized adsorbates-PANI structures, and the average values of NO_3_^−^ and Cl^−^ are presented. **b** Separation factors toward nitrate over chloride and difference in binding energies (BEs) for nitrate and chloride to four different PANI species. **c** A representation of NO_3_^−^ binding to emeraldine salt (ES) form of PANI. The corresponding distances (in Å) between PANI and the adsorbates are presented. Cyan and pink correspond to positive and negative charge density differences, respectively. LS leucoemeraldine salt, ES emeraldine salt, EB emeraldine base, PS pernigraniline salt.

To elucidate the affinity and selectivity of the electrosorption of nitrate on PANI, we carried out density functional theory (DFT) calculations and structure optimization based on classical force fields to investigate the underlying binding mechanism (see “Methods”). Theoretical calculations and experimental results were correlated by selecting six representative PANI-nitrate binding configurations based on our pH-potential heatmap (Fig. 4c, d). First, the DFT binding energy (BE, see Methods for calculation details of the binding energy) was lower (thus more stable) for more reduced salts than for more oxidized salts (e.g., −4.44, −1.88, and −1.41 eV/adsorbate for [LS-nitrate], [ES-nitrate], and [PS-nitrate], respectively) (Supplementary Fig. 12). The trend in BEs for PANI salts is in accordance with the uptake capacity (e.g., 5.2, 71.1, and 30.7 mg NO_3_^−^ g^−1^ PANI, for LS, ES, and PS, respectively), except for LS, where electrostatic repulsion from negative current dominated. Bader charge analysis revealed that nitrate and chloride anion bound to the more reduced PANI species exhibited a more negative charge, indicating stronger interaction with hydrogen bound to nitrogen in PANI (Fig. 5a).

We also compared the BEs of [PANI-nitrate] and [PANI-chloride] and correlated them with the trend in SF determined experimentally. The BE of [LS-chloride] was 0.11 eV/adsorbate smaller than [LS-nitrate], implying higher stability of [LS-chloride] complex (Fig. 5b and Supplementary Fig. 12). Our experimental SF (toward nitrate over chloride) was 0.70, indicating that chloride is more selective and agreeing with DFT calculation (Fig. 5b). On the other hand, ES, EB, PS exhibited lower BE for nitrate compared chloride, and PS exhibited the largest difference between BEs of [PANI-nitrate] and [PANI-chloride], followed by ES and EB. Interestingly, the trend in SFs of four different species—PS (SF: 3.45) > ES (SF: 3.19) > EB (SF: 2.42) > LS (SF: 0.70)—is in agreement with the prediction from the difference in BEs between nitrate and chloride (Fig. 5b). After looking more closely, the adsorption of nitrate was characterized by a shorter distance between nitrate and PANI (Supplementary Fig. 13) than the distance between chloride and PANI (Supplementary Fig. 14), for all PANI species analyzed. Also, structure optimization based on classical force fields revealed the repulsive interaction between adsorbates and PB, resulting in a distance larger than 8 Å from PANI, confirming experimental observations that PB does not significantly bind nitrate. On the other hand, in ES, hydrogen bonding between NO_3_^−^ and benzoid amine (−NH−) occurs, with the distance between nitrate and PANI being 1.74 and 1.80 Å (Fig. 5c). We found that nitrate-PANI interactions increase the −NH− bonds by 0.03–0.06 Å compared to the pristine PANI structures, with the most pronounced effect on LS, providing evidence of electrochemically-activated hydrogen bonding^42,43^. The spectroscopic observations, electrosorption results, and DFT investigation support the selective nature of PANI electrosorbents toward nitrate, with the mechanism being a synergistic combination of electrostatics and hydrogen bonding. It is important to note that accuracy of the binding energies can be improved by leveraging more realistic simulation going forward. For example, first-principles molecular dynamics can be used to explicitly account for solvent effects and applied potentials, as well as ion adsorption event^44,45^. Although more accurate, these approaches are computational very intensive. In this regard, hybrid quantum-classical methods, such as reference interaction site model (RISM)^46^, offer a promising avenue for the description of applied potentials and solvent effects at a smaller computational cost. These approaches will be pursued in future studies.

### Reversible electrochemical release of nitrate and coupled electroconversion into ammonia

**Fig. 6:**
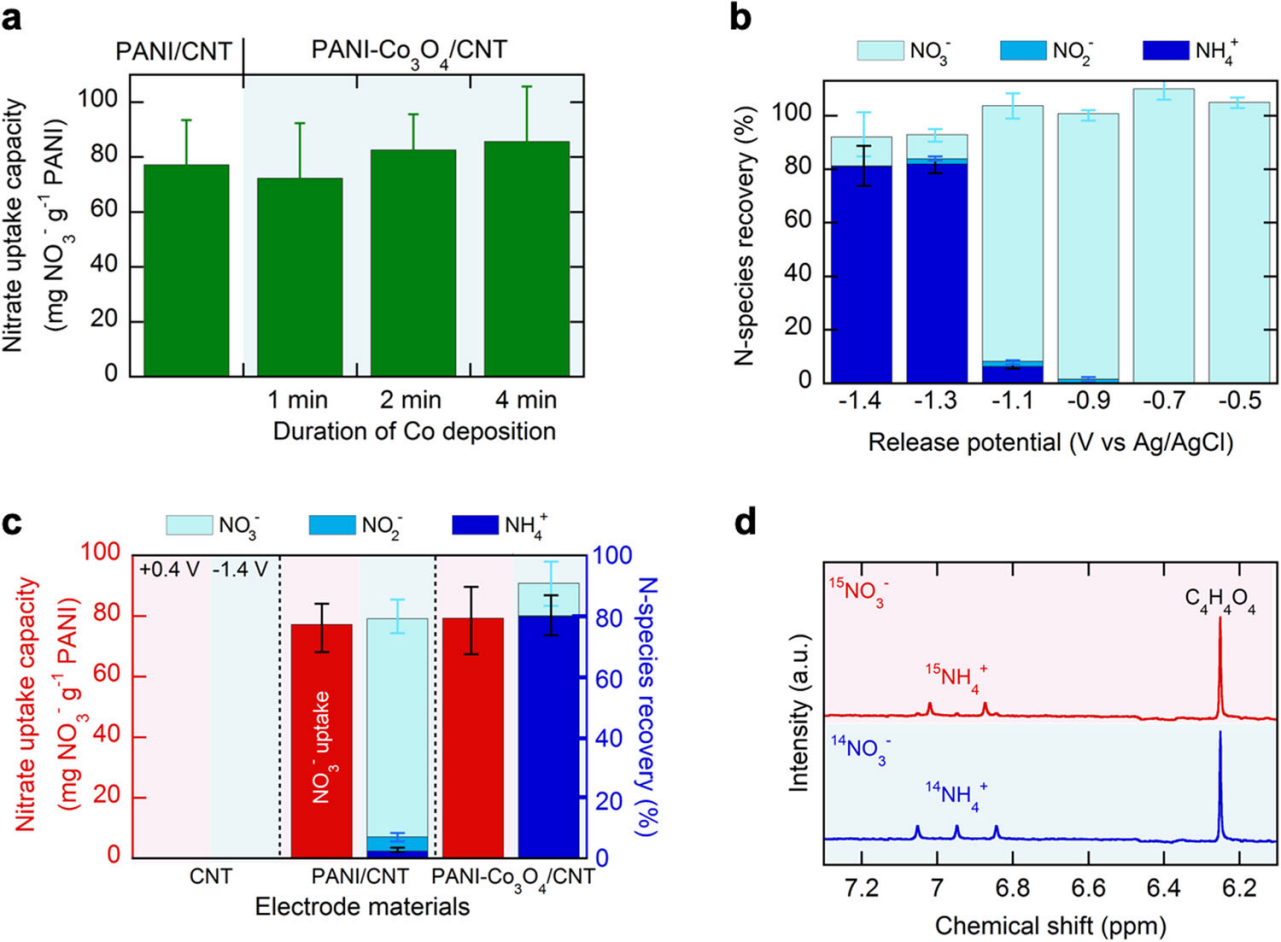
Reactive separations using PANI-Co_3_O_4_/CNT composites. **a** Nitrate uptake capacity using PANI/CNT and PANI-Co_3_O_4_/CNT composites at various Co_3_O_4_ loadings. Electrosorption was carried out in 5 mM NaNO_3_ + 5 mM NaCl at +0.4 V vs Ag/AgCl for 0.5 h. Within the experimental range, the existence of Co_3_O_4_ did not bring about any decrease in nitrate electrosorption. **b** Percentage of nitrogen species recovered in the form of nitrate, nitrite, and ammonium after release into 0.1 M NaCl for 1 h at various electrode potentials. Before release, electrosorption was carried out in 5 mM NaNO_3_ + 5 mM NaCl at +0.4 V vs Ag/AgCl for 0.5 h. **c** Nitrate uptake capacity (left *y* axis) and percentage of nitrogen species recovered (right y axis) after one full cycle of electrosorption and regeneration using CNT, PANI/CNT, and PANI-Co_3_O_4_/CNT electrodes. Electrosorption was carried out in 5 mM NaNO_3_ + 5 mM NaCl at +0.4 V vs Ag/AgCl for 0.5 h, and regeneration was performed in 0.1 M NaCl at −1.4 V vs Ag/AgCl for 1 h. **d** ^1^H-NMR spectra for the regeneration electrolytes after a full cycle of electrosorption (+0.4 V vs Ag/AgCl) and release (−1.4 V vs Ag/AgCl). a.u., arbitrary units. Error bars indicate the standard error of the mean (*n* = 2). See “Methods” for experimental details.

Our preliminary tests with Co_3_O_4_/CNT electrodes demonstrated catalytic activity at a potential below −1.0 V vs Ag/AgCl, with a reasonable ammonia yield rate (566 ug h^−1^ cm^−2^), high faradaic efficiency (87.7%), and high product selectivity (86.8%) at −1.4 V vs Ag/AgCl (Supplementary Fig. 15). Co_3_O_4_ was reported to be stable in acidic media and under oxidizing conditions^30,31^, with these stability properties being highly desirable given the swing to anodic potentials during electrochemical nitrate capture in our work. We tested whether the presence of Co_3_O_4_ interfered with nitrate adsorption in the PANI-Co_3_O_4_/CNT system. Co_3_O_4_ loading was controlled by varying the duration of Co(OH)_2_ deposition (1, 2, and 4 min). Next, we electropolymerized PANI at a constant current (3 mA cm^−2^) in 0.2 M aniline + 0.5 M H_2_SO_4_ for 5 min to achieve a constant loading of PANI (see Supplementary Table 2 for mass loading of PANI and Co_3_O_4_). As shown in Supplementary Fig. 16, the original redox-behavior of PANI was preserved regardless of Co_3_O_4_ loading. Likewise, the presence of clusters of Co_3_O_4_ nanosheets did not influence nitrate uptake capacity, indicating the absence of any noticeable steric inhibition (Fig. 6a). Thus, PANI-Co_3_O_4_/CNTs with Co(OH)_2_ deposition for 4 min were used for further experiments.

Next, we investigated the effect of release potential on the final speciation of nitrogen into a desorption electrolyte. Electrosorption of nitrate was carried out with PANI-Co_3_O_4_/CNT at +0.4 V vs Ag/AgCl in 5 mM NaNO_3_ + 5 mM NaCl, and discharged at various release potentials into a clean electrolyte solution (0.1 M NaCl). Figure 6b shows that close to 100% nitrate recovery was obtained at moderate potentials, including −0.5 and −0.7 V vs Ag/AgCl, demonstrating reversible PANI regeneration. Reduced species, like nitrite and ammonium, started to appear after release at −0.9 V vs Ag/AgCl, and at −1.4 V vs Ag/AgCl >80% of nitrogen was recovered as ammonium (Fig. 6b). As the electrode potential changed from −1.1 to −1.3 V vs Ag/AgCl, ammonia production stepped up, suggesting that at −1.1 V vs Ag/AgCl, activation polarization dominated and mass transport was the limiting factor at −1.3 V vs Ag/AgCl. As a control, CNT did not show any uptake of nitrate, with no nitrogen species recovered or released (Fig. 6c). PANI/CNT without Co_3_O_4_ also showed similar electrosorption performance as PANI-Co_3_O_4_/CNT, but after release at −1.4 V vs Ag/AgCl exhibited <10% conversion to ammonium, due to the absence of catalytically active sites (Fig. 6c). Thus, both Co_3_O_4_ and the PANI/CNT are required to achieve the separation and reaction of nitrate to ammonium in an electro-swing fashion. PANI-Co_3_O_4_/CNT prepared by physically mixing each component exhibited poorer nitrate uptake (<35 mg g^−1^) and nitrate-to-ammonia conversion (<55%), because the agglomeration of each component hindered efficient mass transport (Supplementary Fig. 17). In sum, we enable delicate design approach of dual-function material to achieve modular tuning of the speciation in an efficient nitrogen recovery process in an on-demand basis.

In addition, cycling tests were conducted with the PANI-Co_3_O_4_/CNT electrodes being charged at +0.4 V vs Ag/AgCl for electrosorption, in the presence of 5 mM NaNO_3_ + 5 mM NaCl for 0.5 h, and then discharged at −0.5 or −1.4 V vs Ag/AgCl 1 h into 0.1 M NaCl. In spite of the decrease in uptake capacity after the first cycle, the working capacity of PANI-Co_3_O_4_/CNT was stabilized at 40 mg NO_3_^−1^ g^−1^ PANI over four cycles (Supplementary Fig. 18). When discharged at −0.5 V vs Ag/AgCl, nitrate was the final speciation with >75% recovery for all the four cycles (Supplementary Fig. 18a). When released at −1.4 V vs Ag/AgCl, the conversion efficiency into ammonium declined slightly, but still maintained at >65% after the 4th cycle (Supplementary Fig 18b). No significant cobalt leaching was observed in an inductively coupled plasma assay in the adsorption/release electrolyte (Supplementary Fig. 18). Further, high-resolution XPS analysis revealed that there was no substantial change in Co^3+^/Co^2+^ ratio after single anodic (+0.4 V vs Ag/AgCl) and cathodic (−1.4 V vs Ag/AgCl) charging and after four repeated cycles, indicating stable state of valence of cobalt oxide (Supplementary Fig. 19). While this promising performance serves to demonstrate the proof-of-concept for reactive separation within the current work, the development of more tailored and stable nitrogen reduction catalysts or further materials optimization of nanoparticle-polymer distance can even further increase stability and efficiency.

Finally, we performed isotope-labeling tests through electrosorption at +0.4 V vs Ag/AgCl in the presence of ^14^NO_3_^−^ or ^15^NO_3_^−^, followed by release at −1.4 V vs Ag/AgCl in 0.1 M NaCl (See Methods). ^1^H-NMR spectra of desorption electrolytes (Fig. 6d) revealed that, after release, ^15^NO_3_^−^-adsorbing PANI-Co_3_O_4_/CNT led to a doublet (^15^N-^1^H coupling) while ^14^NO_3_^−^-adsorbing PANI-Co_3_O_4_/CNT produced a triplet (^14^N–^1^H coupling), thereby verifying the origin of ammonia from adsorbed nitrate. In addition, the isotope-labeled quantification was consistent with the colorimetric NH_3_ assay utilizing indophenol blue (Supplementary Fig. 20).

### Integration of separation, up-concentration, and nitrate-to-ammonia conversion from nitrate-impacted agricultural streams

**Fig. 7:**
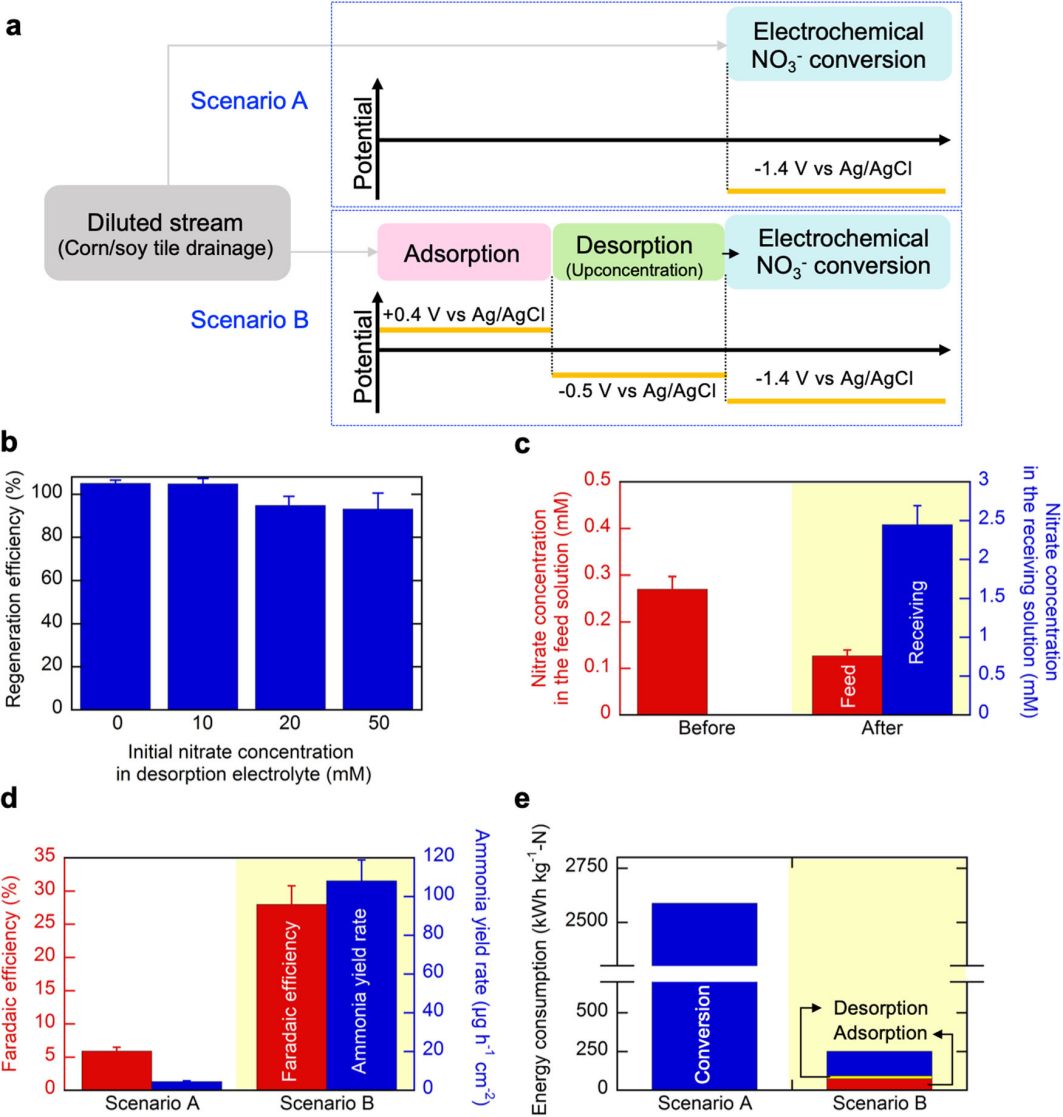
Integration of separation, up-concentration, and conversion and energy analysis. **a** A schematic illustration of two scenarios for treating a dilute nitrate stream (corn/soy tile drainage sample collected from the University of Illinois Energy Farm). In scenario A, an electrocatalyst directly electrocatalyzes the dilute nitrate feed. In scenario B, to overcome the low nitrate concentration and conductivity, a full cycle of adsorption and regeneration was performed to generate up-concentrated, localized nitrate stream, which then can be electrocatalyzed by the same electrode. All the operation can be done in one device, solely by controlling electrode potential. **b** Regeneration efficiency (nitrate recovered/nitrate adsorbed) measured after release at −0.5 V vs Ag/AgCl into 0.1 M NaCl containing various initial nitrate concentrations. Before release, adsorption was carried out in 5 mM NaNO_3_ + 5 mM NaCl at +0.4 V vs Ag/AgCl for 0.5 h. **c** Nitrate concentration of the diluted tile drainage feed and the receiving solution before and after a full cycle of adsorption (in 20 mL) and desorption (in 1 mL) in the scenario B. **d** Faradaic efficiency and ammonia yield rate of PANI-Co_3_O_4_/CNT electrodes in the scenario A and B. **e** Comparison of the energy consumptions (kWh kg^−1^-N) in the scenario A and B. Error bars indicate the standard error of the mean (*n* = 2).

The key challenge of most major nitrate sources (e.g., agricultural groundwater and runoff) is their low concentrations and conductivity, and the presence of natural interfering species. Thus, to understand the role combined effect of nitrate concentration and conductivity on reactive separation and conversion to ammonia in practical water streams, we established two application scenarios (Fig. 7a), and applied them for reactive separations of nitrate from corn/soy tile drainage collected from University of Illinois Energy Farm, in which nitrate concentration and conductivity were 0.27 mM and 505 µS/cm, respectively (see Supplementary Table 3 for the composition of the tile drainage). For scenario A, direct electrochemical reduction of nitrate was carried out in a diluted stream (20 mL of tile drainage). For scenario B, we performed a full cycle of electrosorption (+0.4 V vs Ag/AgCl) and release (−0.5 V vs Ag/AgCl) for nitrate capture and up-concentration, respectively; for the electrosorption, 20 mL of the tile drainage was used, and for desorption, smaller receiving volume (1 mL) of 0.1 M NaCl was used for nitrate up-concentration.

In a preliminary experiment, the composite electrodes released >93% of adsorbed nitrate into a pre-concentrated synthetic nitrate stream (50 mM) (Fig. 7b), proving the feasibility of electrochemical up-concentration. Figure 7c shows the capability of PANI-Co_3_O_4_/CNT transferring nitrate from the tile drainage to the receiving solution, generating a final nitrate concentration of 2.45 mM after a full elctrosorption/desorption cycle (+0.4/−0.5 V vs Ag/AgCl). At the same time, the concentration of nitrate in the feed was reduced to 0.13 mM. The full cycle of electrosorption/regeneration demonstrates the coupling of water remediation, and the generation of localized nitrate streams with the required conductivity for efficient electrocatalytic conversion to ammonia.

Afterward, the up-concentrated nitrate was electrocatalyzed to ammonium by further reducing the potential down to −1.4 V vs Ag/AgCl (Fig. 7a), within the same electrochemical device. The increased nitrate concentration and conductivity in the concentrated receiving solution provided an enhanced electrochemical environment with increased mass transfer, so scenario B exhibited a remarkable enhancement in faradaic efficiency (28%) and ammonia production rate (108.1 ug h^−1^ cm^−2^) compared to directly electrocatalyzing the tile drainage, which exhibited much poorer Faradaic efficiency (5.9%) and ammonia production rate (4.5 ug h^−1^ cm^−2^) (scenario A) (Fig. 7d) due to mass transfer limitations and ionic resistance. Even when the time required for adsorption and desorption were all considered for the determination of ammonia production rate, the revised production rate in scenario B (46.1 ug h^−1^ cm^−2^) was still an order-of-magnitude greater than scenario A. Furthermore, an energy analysis revealed that for scenario B, the total energy consumption (251.3 kWh kg^−1^-N) including adsorption (18.2 kWh kg^−1^-N), desorption (74.0 kWh kg^−1^-N), and catalysis (159.1 kWh kg^−1^-N) still greatly outperformed the scenario A (2589.0 kWh kg^−1^-N), demonstrating that the approach from Scenario B can mitigate the intrinsic critical limitations of directly utilizing dilute nitrate streams (Fig. 7e), and provide a new strategy for nitrate valorization going forward.

**Fig. 8:**
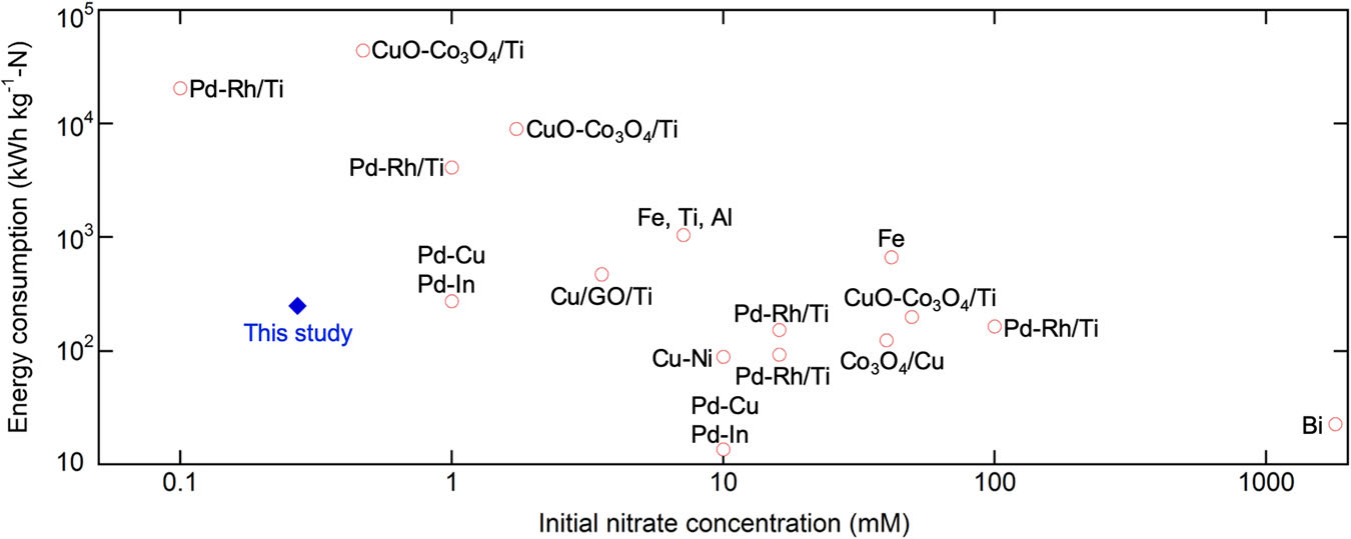
Energy consumption of reported electrochemical nitrate reduction studies. Starting nitrate concentrations and corresponding energy consumptions (kWh kg^−1^-N) in reported electrochemical nitrate reduction studies^20,29,47–53^.

We also carried out a comparative analysis of the specific energy consumption (kWh kg^−1^-N) in our work with reported studies on electrochemical nitrate reduction (Fig. 8)^20,29,47–53^. The starting nitrate concentration was shown to be negatively correlated with specific energy consumption, due to limitation in mass transfer and side reactions^10,51,53^. Compared to other electrocatalytic systems that treat similar concentrations of starting nitrate (0.1–3.5 mM NO_3_^−^ with a specific energy consumption of 275–44,000 kWh kg^−1^-N), our study shows a significant reduction in energy consumption (251.3 kWh kg^−1^-N starting at 0.27 mM), providing an energy-efficient way for treating nitrate (Fig. 8). While direct NH_3_ synthesis using Haber–Bosch process is so far unsurpassed in energy efficiency for global scale synthesis, our study shows advances in nitrate reduction from dilute pollution sources is feasible and provides a promising waste valorization pathway to promote sustainable circularity of nitrogen. Future optimization of electrochemical configurations (flow systems), operational parameters (volume ratio between feed and receiving), and electrochemical operation mode are expected to result in a substantial saving in electrical energy. Going forward, the translation to flow-through systems can provide more practical data, as well as process modeling of multiple tandem electrosorption-based systems to minimize intermittency, with some cells operating in electrosorption-mode and some in release/electroreduction mode. We anticipate further advancements in the design of redox electrodes, where distances between the electrosorbent (polymer) and electrocatalyst (nanoparticle) can be delicately controlled to ensure even higher interfacial accumulations of nitrate. In sum, we envision our coupled reaction-separation concepts can be generalized as a strategy for process intensification, not only for nitrogen valorization, but possibly even other key energy and chemical processes.

In summary, we have successfully demonstrated that the combination of a redox-electrosorbent and an electrocatalyst enabled the energy-efficient reactive separation of dilute nitrate waste streams and valorized them into value-added ammonium. PANI-electrodes displayed selectivity for nitrate by utilizing both hydrogen bonding and ion exchange, with the underlying mechanisms elucidated through both electrosorption experiments and electronic structure calculations. Demonstrating remarkable reversibility and catalytic activity, the composite allowed potential-controlled tuning of nitrogen speciation (~100% nitrate recovery at −0.5 V vs Ag/AgCl or >80% conversion to ammonium at −1.4 V vs Ag/AgCl), in addition to providing modular electrochemically-mediated up-concentration. The PANI-Co_3_O_4_/CNT composite was applied for the combined reactive separation of dilute nitrate from a practical agricultural tile drainage, which demonstrated 24- and 10-fold increase in ammonia production rate (108.1 ug h^−1^ cm^−2^) and energy efficiency (251.3 kWh kg^−1^-N) as compared to reaction only (4.5 ug h^−1^ cm^−2^ and 2589.0 kWh kg^−1^-N), outperforming other electrocatalytic systems that have been evaluated at similarly low nitrate concentrations. From a nitrate remediation perspective, our study provides a highly efficient, chemical-free option which couples the benefit of ammonia generation. From an ammonia synthesis/waste valorization perspective, our approach enables the direct utilization of dilute nitrate as a feedstock for decentralized ammonia production, potentially mitigating the need for carbon-intensive Haber–Bosch process, and establishing new integrated pathways for chemical feedstock or energy carrier production. Fundamentally, our work highlights the central importance of integration of reaction and separations in electrochemical conversion, and provide a generalized strategy for reactive separation of dilute molecules through overcoming intrinsic transport limitations by selective electrosorption.

## Methods

### Preparation of PANI-Co_3_O_4_/CNT electrodes

As a substrate, a titanium (Ti) mesh (Fuel Cell Store, thickness: 2 inch, strand width: 0.004 inch, percent open area: 62%) was cut into a 1.5 cm × 2 cm. 18 mg carbon nanotubes (multiwalled carbon nanotubes, Sigma-Aldrich) were dispersed in 3 mL of N,N-dimethylformamide (DMF) by sonicating for 3 h in icy water. The titanium meshes were coated with the CNT slurry by dip-coating, and an average CNT loading of 1.5 mg cm^−2^ was achieved. The fabrication route of PANI-Co_3_O_4_/CNT is shown in Supplementary Fig. 1. Co_3_O_4_/CNT electrodes were prepared by Co(OH)_2_ electrodeposition followed by heat treatment. The electrodeposition was performed in 0.1 M Co(NO_3_)_2_·6H_2_O at a fixed potential of −1.0 V vs Ag/AgCl using CNT-coated Ti as a working electrode. Unless otherwise stated, electrodeposition lasted for 4 min. Thus-obtained electrodes (Co(OH)_2_/CNT) were rinsed with deionized water, and dried in an oven at 100 °C. The Co(OH)_2_/CNT electrodes were subsequently heated at 200 °C for 1 h with a heating rate of 3 °C min^−1^ to obtain Co_3_O_4_/CNT. The electropolymerization of PANI was performed under galvanostatic conditions in 0.2 M aniline + 0.5 M H_2_SO_4_, using as-prepared Co_3_O_4_/CNT as an anode. Unless otherwise indicated, electrodeposition was carried out at 3 mA cm^−2^ for 5 min. After the electropolymerization, the obtained electrodes were washed with deionized water. The active area of the electrode was 1.5 cm^2^. For PANI/CNT and Co_3_O_4_/CNT, we utilized a simple gravimetric method to determine the mass difference between before and after electrodeposition of PANI or Co_3_O_4_. For PANI-Co_3_O_4_/CNT composites, a gravimetric method was used to measure the sum of the mass of PANI and Co_3_O_4_. Following digestion, inductively coupled plasma optical emission spectrometry (ICP-OES) analysis was used to estimate the cobalt content, from which PANI content was determined.

### Electrochemical testing

Electrochemical tests were conducted with a potentio/galvanostat (Squidstat Solo, Admiral Instrument). Electrosorption of nitrate was conducted in a BASi (Bioanalytical Systems, Inc.) VC-2 voltammetry electrochemical cell with a PANI/CNT or PANI-Co_3_O_4_/CNT working electrode, a platinum wire (length: 7.5 cm, diameter: 0.5 mm, purity: 99.99%) as a counter electrode, and Ag/AgCl (3 M NaCl) as a reference electrode. An electrolyte containing 3 mL of 5 mM NO_3_^−^ and 5 mM Cl^−^ was used, with the pH of the solution being controlled with HCl or NaOH. The potentiostatic electrosorption was performed to evaluate the performance of PANI electrosorbent at various oxidation states, at 0, +0.2, +0.4, +0.6, and +0.8 V vs Ag/AgCl. Unless otherwise specified, electrosorption was performed for 30 min. The cell resistance of the adsorption cell was found to be 193 ± 5 Ω.

PANI-Co_3_O_4_/CNT were regenerated in either BASi VC-2 cells (31 ± 1 Ω, Supplementary Fig. 21) or a H-type cell (24 ± 1 Ω, Supplementary Fig. 22) using the Nafion 117 membrane (DuPont). When using the VC-2 cells for regeneration, the counter electrode was isolated from the working electrolyte by the use of a glass body and porous CoralPor™ tip to avoid oxidation of produced ammonium. To prepare Nafion 117 for H-type cell tests, Nafion sheets were cut and boiled in 3% hydrogen peroxide for 1 h, rinsed in deionized water, boiled for 2 h in deionized water to remove residual peroxide, treated in 0.5 M H_2_SO_4_ for 1 h, and then rinsed and stored in deionized water. Nitrate-adsorbing electrodes were transferred to a desorption electrolyte of 0.1 M NaCl, and the release (desorption) of nitrate and coupled electrocatalysis were assessed at the following applied potentials: −0.5, −0.7, −0.9, −1.1, −1.3, and −1.4 V vs Ag/AgCl. Unless otherwise noted, electrochemical reductions were conducted for 1 h. For all the electrochemical tests, the potential was not IR-compensated.

### Quantification methods

For colorimetric analysis, a UV–vis spectrophotometer (Cary 60, Agilent) was employed. Nitrate was determined using salicylic acid method^54^. In total, 100 µL of the sample was taken and then 400 µL of 5% (w/v) salicylic acid in concentrated sulfuric acid was added. The mixture was vortexed, and then incubated for 20 min at room temperature. Then, 9.5 mL of 8% (w/v) NaOH solution was added and incubated for 30 min at room temperature. Absorbance at 410 nm was measured, and the same procedure was repeated for building up a calibration curve using several standard solutions (Supplementary Fig. 23).

Nitrite concentration was determined by means of the Griess reagent^55^, which was prepared by mixing equal volumes of a) 10 mg mL^−1^ sulfanilamide + 1.2 M HCl and b) 1 mg mL^−1^ of N-(1-naphthyl)ethylenediamine dihydrochloride. 0.1 mL of an electrolyte sample was mixed with 3.9 mL of deionized water. The mixture of the 4 mL sample and 0.4 mL Griess reagent was incubated for 30 min at room temperature. The absorbance was measured at 540 nm. A calibration curve was constructed using the same method (Supplementary Fig. 24).

For the measurement of ammonium, the indophenol blue method was employed^56^. There were three reagents prepared, including (a) oxidation solution of 0.75 M sodium hydroxide in 10 ml sodium hypochlorite solution (available chlorine 4.00–4.99%), b) coloring solution of 0.4 M sodium salicylate +0.32 M sodium hydroxide, and (c) catalyst solution of 0.1 g sodium nitroferricyanide dihydrate in 10 ml deionized water. 0.3 mL of an electrolyte sample was mixed with 3.7 mL of 0.2 M NaOH. Next, 50 μL of a) oxidizing solution, 500 μL of (b) coloring solution, and 50 μL of c) catalyst solution were sequentially added. After 2 h, the absorbance was measured at a wavelength of 665 nm. To create a calibration curve, the same procedure was applied to several standard solutions (Supplementary Fig. 25).

Nitrate and chloride concentrations were also analyzed using ion chromatography (Dionex Integrion, Thermo) with an autosampler (AS-DV). All samples were adjusted to a pH between 6 and 8, and then diluted 20 times with deionized water. To prepare concentration-peak area curves for calibration, standard solutions of nitrate and chloride (0.1, 0.2, 0.3, 0.4, and 0.5 mM) were prepared. An anionic column (Dionex™ IonPac™ AS22 IC Columns) was used at 35 °C with an eluent flow rate of 1.2 mL min^−1^. An anionic eluent used in this experiment consisted of 4.5 mM Na_2_CO_3_ with 1.4 mM NaHCO_3_. The retention time was set to 15 min and peaks of chloride and nitrate were assigned at 4.5 and 7.2 min, respectively.

Based on the quantification of the aqueous concentrations, the separation factor (α) of PANI toward nitrate over chloride was determined as follows:

$${\alpha }_{{{{{{\rm{nitrate}}}}}},{{{{{\rm{chloride}}}}}}}=\,{\left(\frac{{N}_{{{{{{\rm{nitrate}}}}}},{{{{{\rm{ads}}}}}}}/{N}_{{{{{{\rm{chloride}}}}}},{{{{{\rm{ads}}}}}}}}{{N}_{{{{{{\rm{nitrate}}}}}},{{{{{\rm{sol}}}}}}}/{N}_{{{{{{\rm{chloride}}}}}},{{{{{\rm{sol}}}}}}}}\right)}_{{{{{{\rm{eq}}}}}}}$$

where ${N}_{{{{{{\rm{X}}}}}},{{{{{\rm{ads}}}}}}}$ is the number of moles of X adsorbed by PANI at equilibrium, ${N}_{{{{{{\rm{X}}}}}},{{{{{\rm{sol}}}}}}}$ is the number of moles of X remaining in electrolyte at equilibrium. Also, faradaic efficiency for ammonia synthesis was measured as follows:

$${{{{{\rm{Faradaic\; efficiency}}}}}}=\frac{8\times M\times F}{{Q}_{{{{{{\rm{total}}}}}}}}\times 100$$

where *M* (mol) is the amount of NH_3_ produced, *F* is the Faraday constant (96,485 C mol^−1^), *Q*_total_ is the total charge passed. Considering that eight electrons are involved in nitrate-to-ammonia conversion, *n* = 8 was used for the determination of faradaic efficiency.

### Determination of point of zero charge

The point of zero charge (PZC) was determined using a batch equilibrium test to determine the surface acid/base transition properties of PANI/CNT. The electrodes used for PZC measurements were electropolymerized in 0.2 M aniline + 0.5 M H_2_SO_4_ at a constant current of 3 mA cm^−2^ for 20 min. As a next step, leucoemeraldine, emeraldine, and pernigraniline PANI/CNT films were formed by applying 0, +0.4, and +0.8 V vs Ag/AgCl for 20 min, respectively. On the basis of a previous paper^38^, the characteristic colors of emeraldine (green) and pernigranline (violet) were confirmed. Afterward, each electrode (about 3.3 mg of PANI) was added to a series of vials containing 3 mL of 10 mM NaCl solution. HCl and NaOH were used to control the initial pH values. The vials were sealed and agitated using a stir bar at 400 rpm for 24 h, after which the pH values of the final solution were determined. PZC value was determined from initial pH vs delta pH plot (Supplementary Fig. 9).

### ^15^N isotope-labeling experiment and ^1^H nuclear magnetic resonance (NMR) analysis

^1^H-NMR characterization of the ^14^NO_3_^−^ and ^15^NO_3_^−^ isotope-tracing experiments was conducted using a Carver B500 Bruker Avance III HD NMR Spectrometer (500 MHz). Before NMR tests, electrosorption was performed using PANI-Co_3_O_4_/CNT electrodes at +0.4 V vs Ag/AgCl in an electrolyte containing (i) ^14^NO_3_^−^ (5 mM Na^14^NO_3_ + 5 mM NaCl) or (ii) ^15^NO_3_^−^ (5 mM Na^15^NO_3_ + 5 mM NaCl). After electrosorption, nitrate-loaded electrodes were transferred into 0.1 M NaCl and regenerated at −1.4 V vs Ag/AgCl for 1 h. Afterward, the release electrolyte was diluted 10 times with 0.1 M H_2_SO_4_ for analysis within suitable concentration range. NMR samples were prepared by mixing 0.5 mL of the diluted solution and 0.1 mL of DMSO-*d6* containing 0.004 wt% (0.41 mM) maleic acid (C_4_H_4_O_4_). For standard solutions, 0.1, 0.2, 0,3, 0.4, and 0.5 mM of ^15^NH_4_Cl were prepared in an equivalent matrix. All NMR measurements were carried out with water suppression and 500 scans.

### Materials characterizations

Powder X-ray diffraction (PXRD) measurements were performed on a Rigaku Miniflex 600, and XRD patterns were compared with references from ICSD (International Crystal Structure Database). The surface valance of the PANI-Co_3_O_4_/CNT before and after use was examined by X-ray photoelectron spectroscopy (XPS, Thermo Scientific K-Alpha) with monochromatic Al K-Alpha source gun. Casa XPS software (UIUC license) was used to analyze XPS spectra. The surface morphology and structure of the prepared samples were analyzed with SEM (Hitachi S4800) operated at an accelerating voltage of 20 kV. TEM and high-resolution TEM (HR-TEM) images were acquired with a Thermo Fisher Themis-Z TEM operated at 300 kV. Energy-dispersive X-ray spectroscopy (EDS) analysis was conducted in the scanning transmission electron microscopy (STEM) mode with Super X-EDS system installed on the same TEM with acceleration voltage of 80 kV. Electron energy loss spectroscopy (EELS) analysis was performed with Gatan Quantum ER/965 GIF with Ultrafast Dual EELS in the same TEM operated at 80 kV.

### Computational details

The polymer unit cells of leucoemeraldine, emeraldine, and pernigraniline bases and salts composed of four elementary units were created using the Avogadro package^57^. First, classical MMFF94s force fields were employed to pre-optimize the atomic structures and identify key binding sites for the adsorbates (NO_3_^−^ and Cl^−^). Specifically, geometry optimization was performed using steepest descent algorithm until a convergence threshold of 10^−8^ eV/Å is reached. The obtained structures were then used to create repetitive cells of PANI, *a* × *b* × *c* Å^3^, where *a* is the optimized cell dimension of the aligned polymer, while *b* and *c* were chosen to be 25 Å to minimize interactions between periodic images. The pre-optimized structures were then used to carry out structural optimizations within the DFT-based plane-wave code Vienna Ab Initio Simulation Package (VASP)^58,59^. The Perdew−Burke−Ernzerhof (PBE) functional under the generalized gradient approximation (GGA) was used to treat exchange-correlation interactions^60^. A cutoff energy of 500 eV was employed in all DFT calculations. Also, the convergence criteria for the energy and atomic forces during structural optimization were 10^−6^ eV and 0.02 eV/Å, respectively. Solvation effects were described using an implicit solvation model that requires a significantly lower computational cost compared to explicit molecular dynamics simulations, in alignment with previous computational studies on polyaniline^61^. To account for the effects of an applied potential, binding energies were computed using PANI structures associated with a specific potential range. In addition, we assume that the binding energies between an ion and PANI varies linearly with applied potential, and therefore the relative binding energy between Cl^−^ and NO_3_^−^ is independent of applied potentials for a given PANI structure. The implicit solvent model implemented in VASPsol^62,63^ was used in all simulations.

The free energies of Cl_2_, NO_3_^−^ were obtained by considering the isolated species in a vacuum box of 15 × 16 × 17 Å^3^. Zero-point vibrational energies and entropic contributions were added as follows:

$$\triangle {G}_{{{{{{{\rm{Cl}}}}}}}_{2}}=\,{E}_{{{{{{{\rm{Cl}}}}}}}_{2}}+{ZPVE}({{{{{{\rm{Cl}}}}}}}_{2})-{{TS}}_{{{{{{{\rm{Cl}}}}}}}_{2}}$$

$$\triangle {G}_{{{{{{{\rm{NO}}}}}}}_{3}^{-}}=\,{E}_{{{{{{{\rm{NO}}}}}}}_{3}^{-}}+{ZPVE}({{{{{{\rm{NO}}}}}}}_{3}^{-})-{{TS}}_{{{{{{{\rm{NO}}}}}}}_{3}^{-}}$$

Here, following previously reported strategies^64–66^, the free energy of Cl^−^ is calculated as:

$$\triangle {G}_{{{{{{{\rm{Cl}}}}}}}^{-}}=\,{\frac{1}{2}G}_{{{{{{{\rm{Cl}}}}}}}_{2}}$$

The binding energies between PANI and the adsorbates were calculated as:

$${\triangle E}_{*{{{{{\rm{ads}}}}}}}=\,\left(\frac{{E}_{*{{{{{\rm{ads}}}}}}}-{E}_{*}-n\triangle {G}_{{{{{{\rm{ads}}}}}}}}{n}\right)$$

where ${E}_{*{{{{{\rm{ads}}}}}}}$ is the DFT energy of PANI in the presence of an adsorbate (Cl^−^, NO_3_^−^), ${E}_{*}$ is the DFT energy of the pristise structure (base/salt), $\triangle {G}_{{{{{{\rm{ads}}}}}}}$ is the free energy of Cl^−^ and NO_3_^−^, *n* is the number of adsorbed species. The Bader charge analysis was performed using the scripts developed by Henkelman et al.^67^.

## Supplementary information

Supplementary Information

## Data Availability

All experimental data reported in this study and Supplementary Information are available from the corresponding author upon reasonable request.
