# Supplementary material for: Coupling nitrate capture with ammonia production through bifunctional redox-electrodes
Source: Nat Commun. 2023 Feb 14;14:823. doi: 10.1038/s41467-023-36318-1 (PMC9929237; doi:10.1038/s41467-023-36318-1)
Supplement: Supplementary file 1 — Supplementary Information [file 41467_2023_36318_MOESM1_ESM.docx]

Supplementary Information

**Coupling nitrate capture with ammonia production through bifunctional redox-electrodes** **Author list**

Kwiyong Kim^1^, Alexandra Zagalskaya^2,3^, Jing Lian Ng^1^, Jaeyoung Hong^1,4^, Vitaly Alexandrov,^3,5^ Tuan Anh Pham^2,6^, Xiao Su^1^*

**Affiliation**

^1^ Department of Chemical and Biomolecular Engineering, University of Illinois at Urbana-Champaign, Urbana, Illinois 61801, United States. E-mail: [x2su@illinois.edu](mailto:x2su@illinois.edu)

^2^ Quantum Simulations Group, Materials Science Division, Lawrence Livermore National Laboratory, Livermore, California 94550, United States

^3^ Department of Chemical and Biomolecular Engineering, University of Nebraska-Lincoln, Lincoln, Nebraska 68588, United States

^4^ Department of Materials Science and Engineering, University of Illinois at Urbana-Champaign, Urbana, Illinois 61801, United States

^5^ Nebraska Center for Materials and Nanoscience, University of Nebraska-Lincoln, Lincoln, Nebraska 68588, United States

^6^ Laboratory for Energy Applications for the Future (LEAF), Lawrence Livermore National Laboratory, Livermore, California 94550, United States

Present address for Dr. Kwiyong Kim: Department of Urban and Environmental Engineering, Graduate School of Carbon Neutrality, Ulsan National Institute of Science and Technology (UNIST), 50 UNIST-gil, Eonyang-eup, Ulju-gun, Ulsan, 44919, Republic of Korea


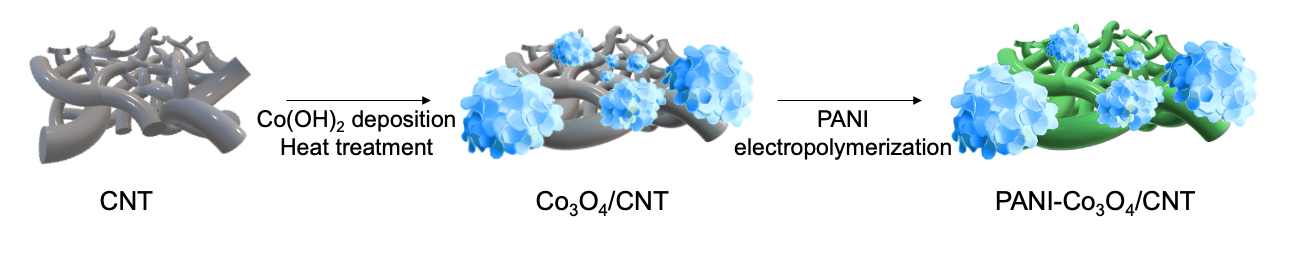


**Supplementary Figure 1.** A process of preparing PANI-Co_3_O_4_/CNT electrodes. See Methods for fabrication details.


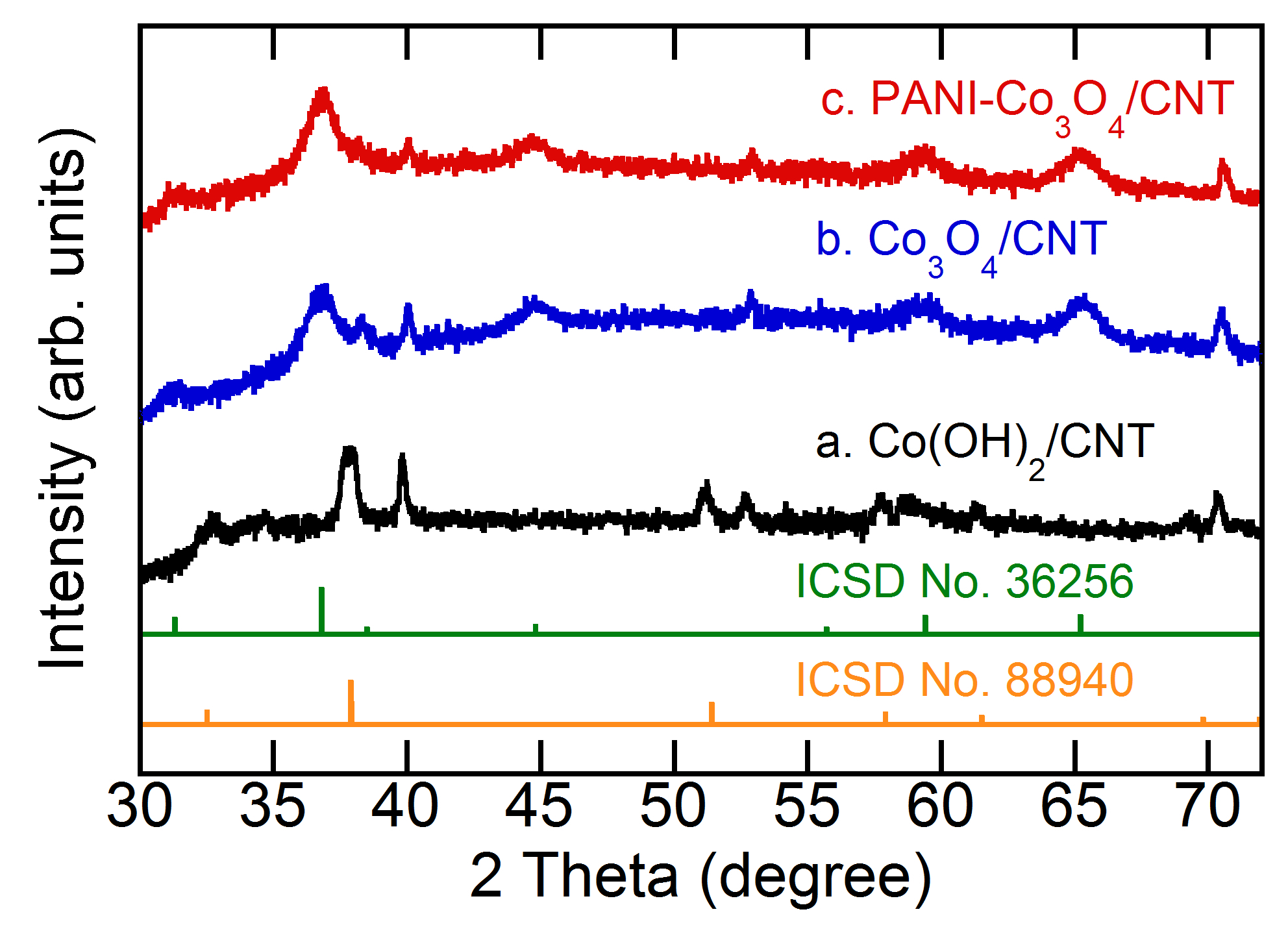


**Supplementary Figure 2.** XRD Patterns of (a) Co(OH)_2_/CNT, (b) Co_3_O_4_/CNT, and (c) PANI-Co_3_O_4_/CNT electrodes. a) Co(OH)_2_/CNT exhibits characteristic peaks at 32.5°, 37.9°, 51.4°, 57.9°, 61.5°, and 69.5°, which are well correlated with (100), (101), (102), (110), (111), and (103) crystal planes of Co(OH)_2_ (ICSD No. 88940). b) Co(OH)_2_ was converted into Co_3_O_4_ after heat treatment for 1 h, as demonstrated by the characteristic peaks at 31.3°, 36.9°, 38.6°, 44.9°, 59.5°, and 65.4° that are indexed to the (220), (311), (222), (400), (511), and (440) lattice planes of Co_3_O_4_ (ICSD No. 36256). c) Electropolymerizing PANI in acidic conditions did not alter the XRD pattern of Co_3_O_4_.

**Supplementary Figure 3.** TEM images of (a) pristine CNT and (b) PANI-coated CNT. In (b), PANI was electropolymerized in 0.2 M aniline + 0.5 M H_2_SO_4_ at a constant current of 3 mA cm^-2^ for 5 min. Scale bars are 20 nm.

**Supplementary Figure 4.** a) A TEM image of PANI-coated CNT. b) A STEM image of PANI-coated CNT. c–d) EDS mapping images for atomic distribution of (c) carbon and (d) nitrogen from STEM image in (b). PANI was electropolymerized in 0.2 M aniline + 0.5 M H_2_SO_4_ at a constant current of 3 mA cm^-2^ for 5 min. Scale bars are 50 nm.


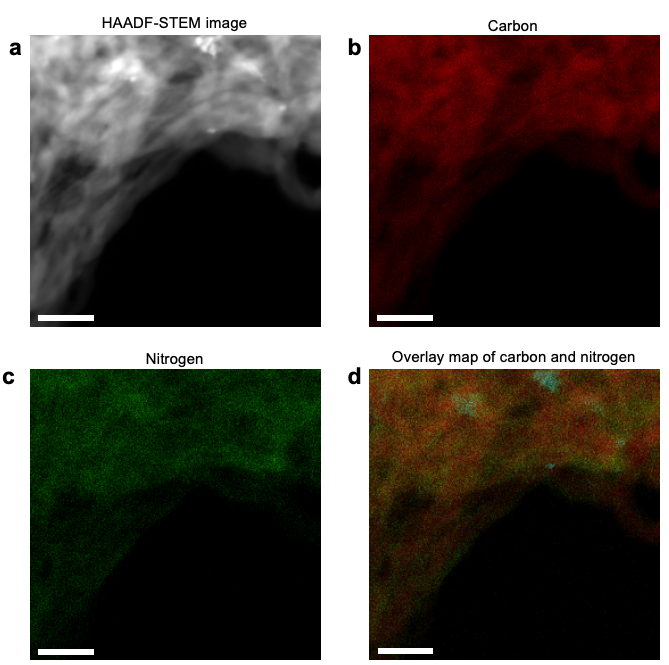


**Supplementary Figure 5.** a) A STEM image of PANI-Co_3_O_4_/ CNT. b–d) EDS mapping images for atomic distribution of (b) carbon, (c) nitrogen from STEM image in (a). d) An overlay map of carbon and nitrogen. PANI was electropolymerized in 0.2 M aniline + 0.5 M H_2_SO_4_ at a constant current of 3 mA cm^-2^ for 5 min. Scale bars are 50 nm.

**Supplementary Figure 6.** SEM images of (a) pristine CNT and (b) PANI-coated CNT. PANI was electropolymerized in 0.2 M aniline + 0.5 M H_2_SO_4_ at a constant current of 3 mA cm^-2^ for 5 min. Scale bars are 1 μm.

**Supplementary Figure 7.** A SEM image of PANI-Co_3_O_4_/CNT composite. Co(OH)_2_ was electrodeposited in 0.1 M Co(NO_3_)_2_ solution at -1.0 V vs Ag/AgCl for 4 min, then converted into Co_3_O_4_ by heat treatment at 200°C for 1 h. Finally, PANI was electropolymerized in 0.2 M aniline + 0.5 M H_2_SO_4_ at a constant current of 3 mA cm^-2^ for 5 min. Scale bar is 5 μm.


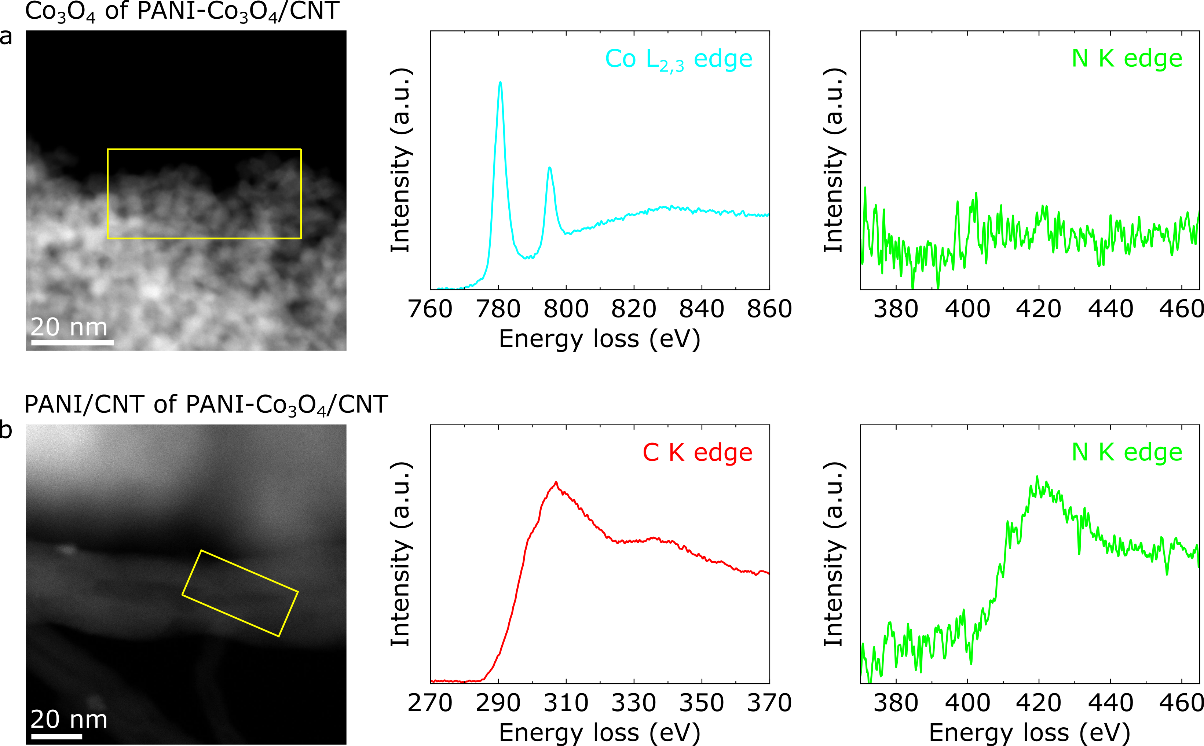


**Supplementary Figure 8.** STEM images of (a) Co_3_O_4_ nanoparticles and (b) PANI/CNT in PANI-Co_3_O_4_/CNT and corresponding EELS spectra acquired from the area marked with yellow boxes. a) Energy loss in Co L_2,3_ edge and N K edge energy range shows clear cobalt peaks while nitrogen signal is undetectable. b) Spectra showing C K edge and N K edge energy range confirm that PANI coexists with CNT. a.u., arbitrary units.

**Supplementary Figure 9.** The change in pH of 10 mM NaCl solution after equilibration with PANI/CNT composites. Initial pH was adjusted using HCl and NaOH. Point of zero charges (PZC) of (a) leucoemeraldine, (b) emeraldine, and (c) pernigraniline were determined to be 5.64, 4.20, and 3.60, respectively. See Methods for details.


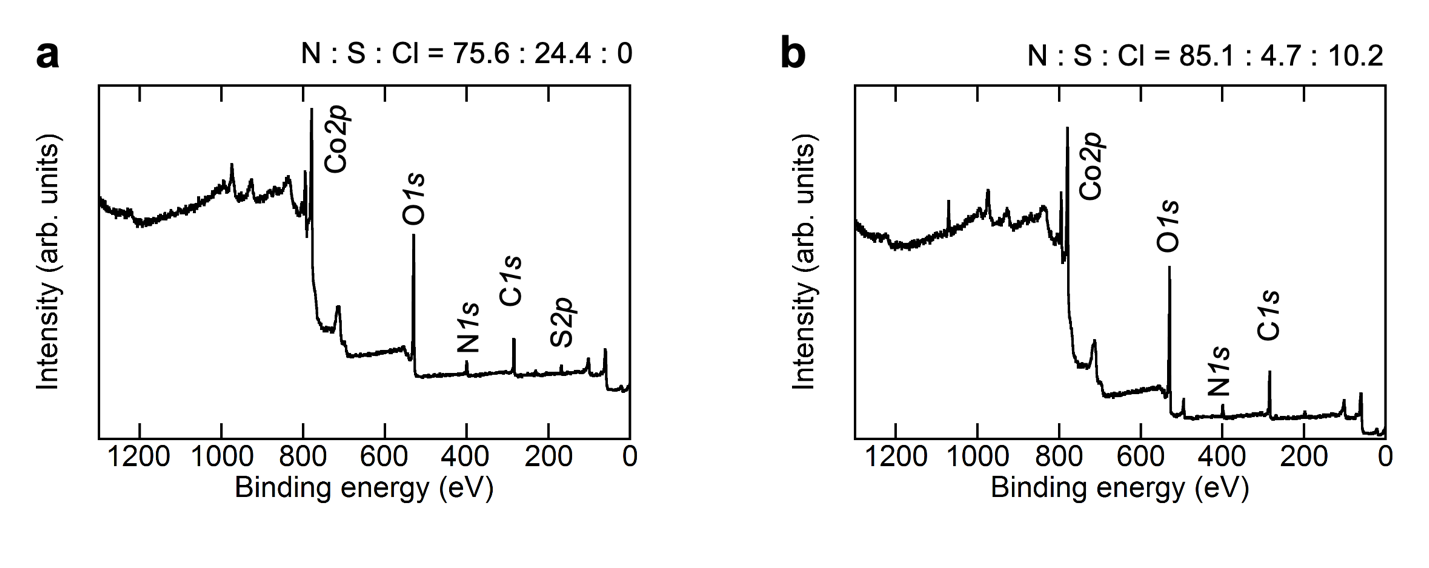


**Supplementary Figure 10.** XPS survey scan for (a) pristine PANI/CNT and (b) PANI/CNT electrode after electrosorption at +0.4 V vs Ag/AgCl in 5 mM NaNO_3_ + 5 mM NaCl.


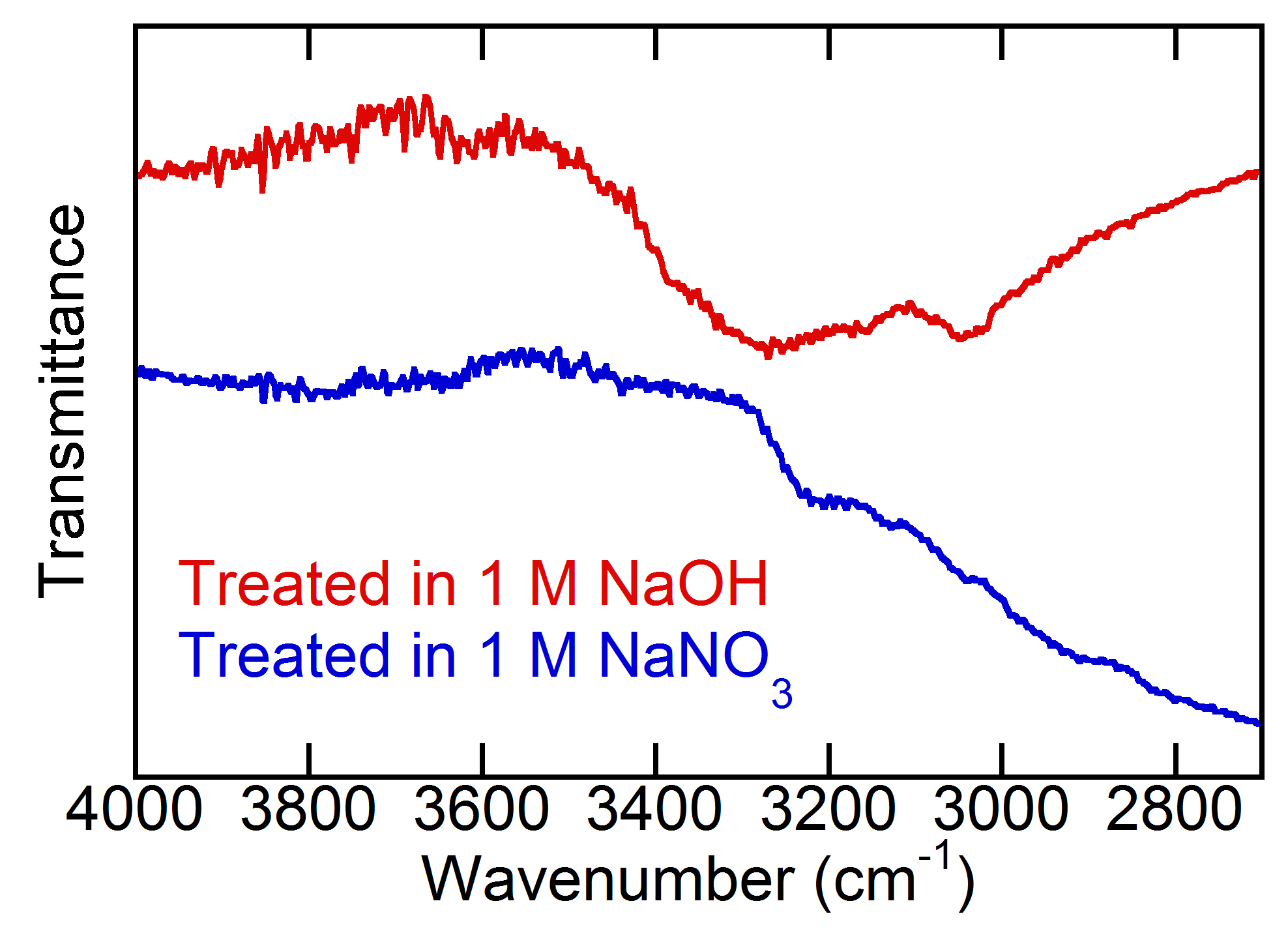


**Supplementary Figure 11.** Attenuated total reflection infrared (ATR-IR) spectroscopy of emeraldine PANI treated in 1 M NaOH (red) and 1 M NaNO_3_ (blue) for 24 h. PANI in emeraldine salt form was synthesized using ammonium persufate as a chemical oxidant in 0.5 M sulfuric acid^1^.


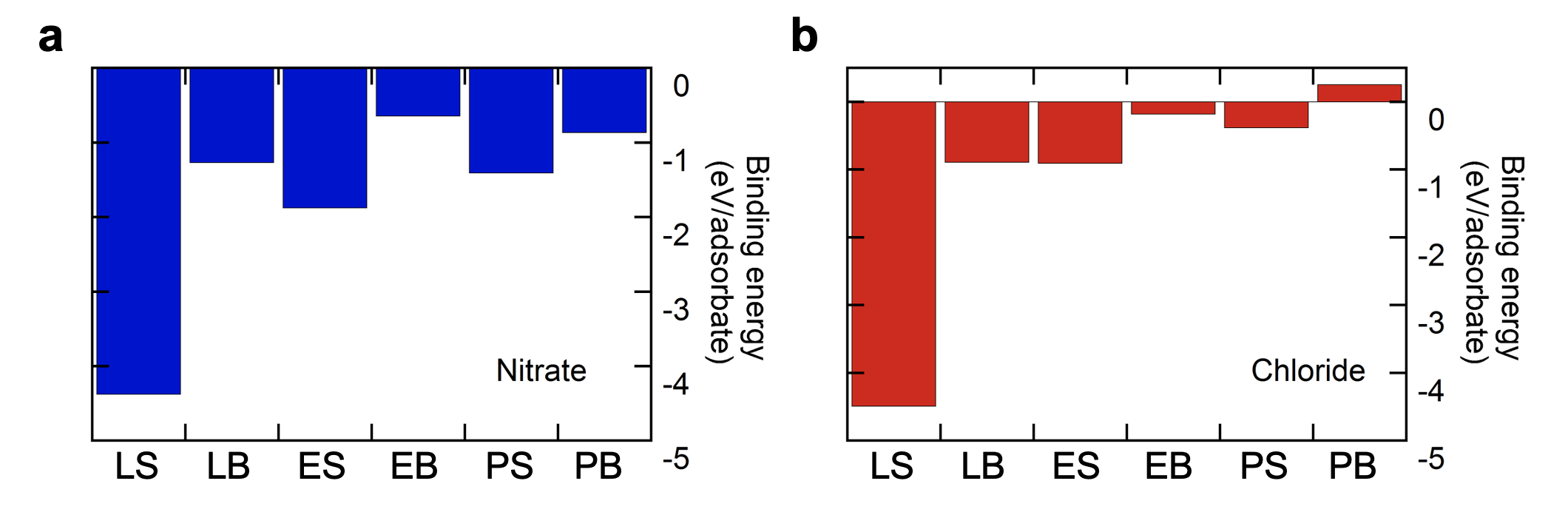


**Supplementary Figure 12.** Binding energy to six different forms of PANI (LS, LB, ES, EB, PS, PB) for (a) nitrate and (b) chloride.


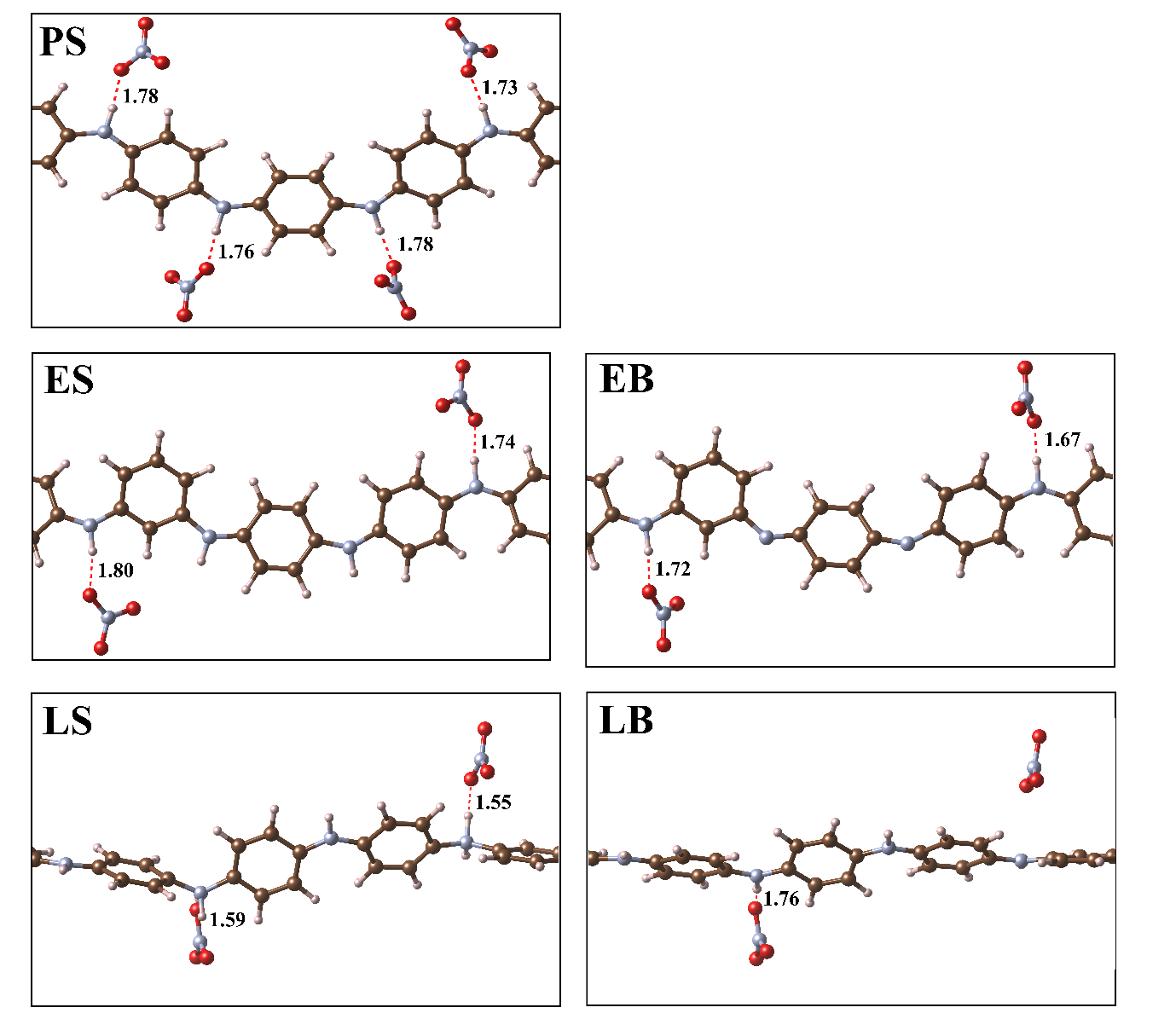


**Supplementary Figure 13.** Schematic of NO_3_^-^ adsorption for different PANI configurations. The distances between NO_3_^-^ and hydrogen atoms are shown in Å. For LB, one of the adsorbates appeared not to bond to the PANI with the corresponding distance of more than 3 Å, therefore it was omitted in the figure. The detailed coordinates of the structures are provided in the Supplementary Table 4.


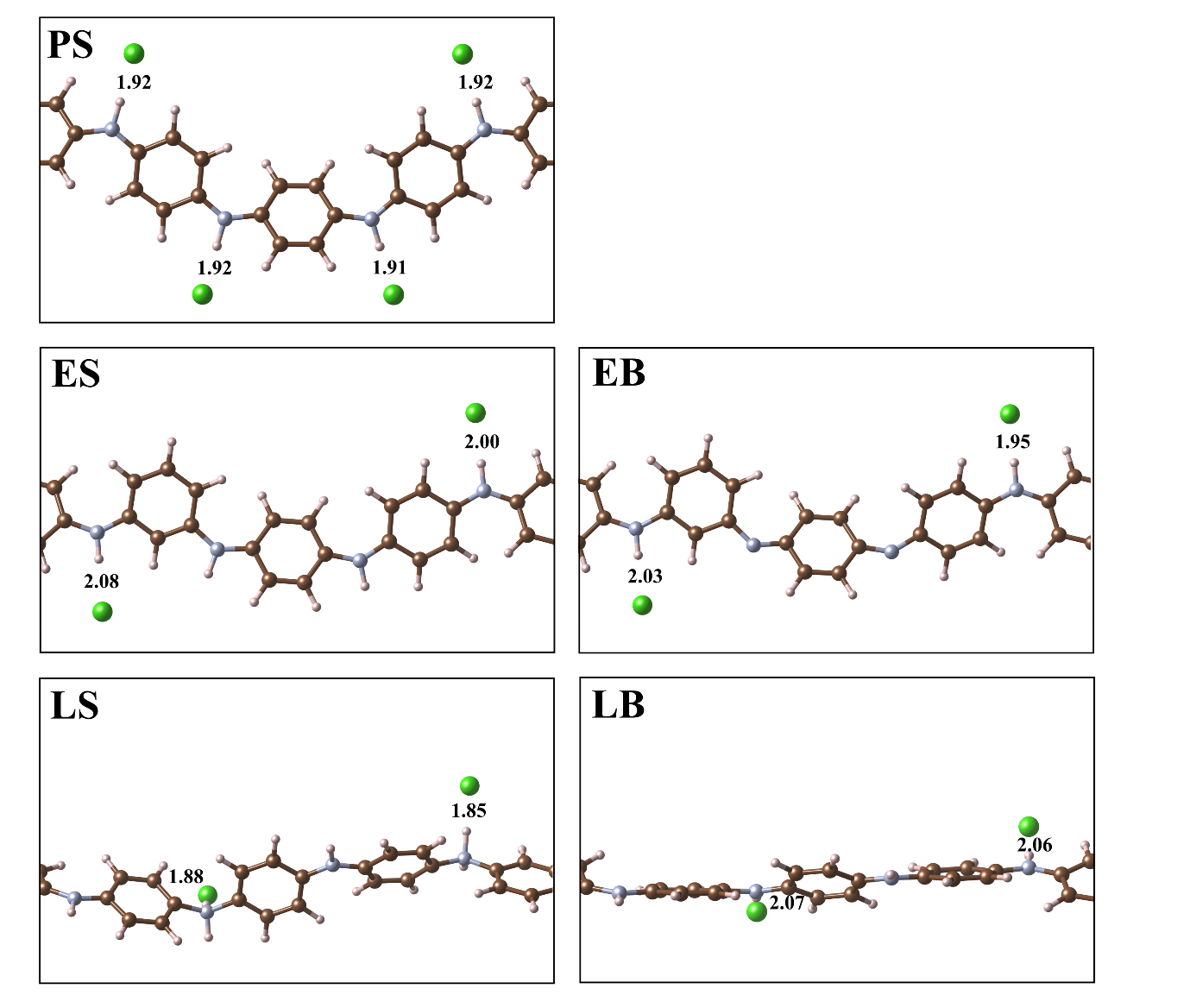


**Supplementary Figure 14.** Schematic of Cl^-^ adsorption for different PANI configurations. The distances between Cl^-^ and hydrogen atoms are shown in Å. The detailed coordinates of the structures are provided in the Supplementary Table 5.


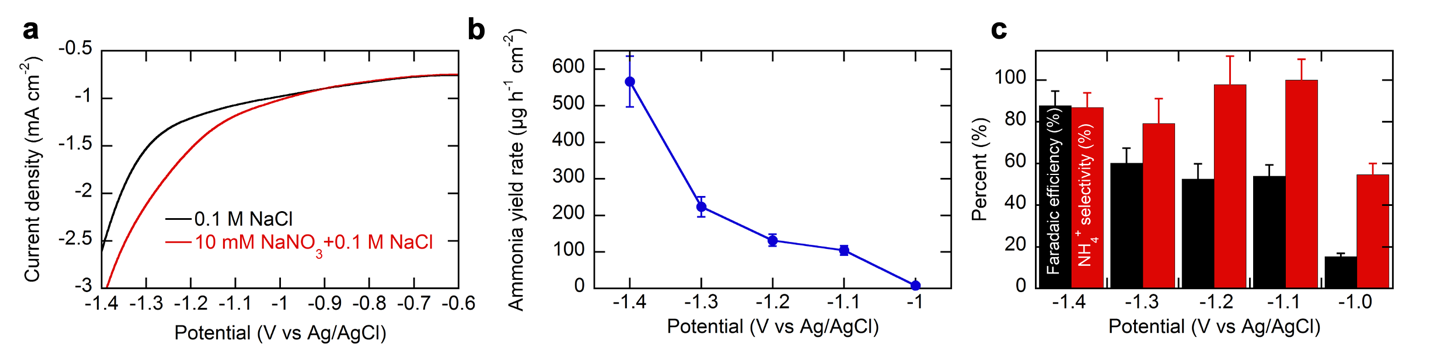


**Supplementary Figure 15.** a) Linear sweep voltammogram of Co_3_O_4_/CNT electrodes without and with 10 mM NaNO_3_ in 0.1 M NaCl. A scan rate of 10 mV s^-1^ was used. b) Ammonia yield rate after chronoamperometric electrocatalysis for 30 min at various potentials and c) corresponding faradaic efficiency and product selectivity. For (b) and (c), the electrolyte was 10 mM NaNO_3_ + 0.1 M NaCl. Error bars indicate the standard error of the mean (n = 2).


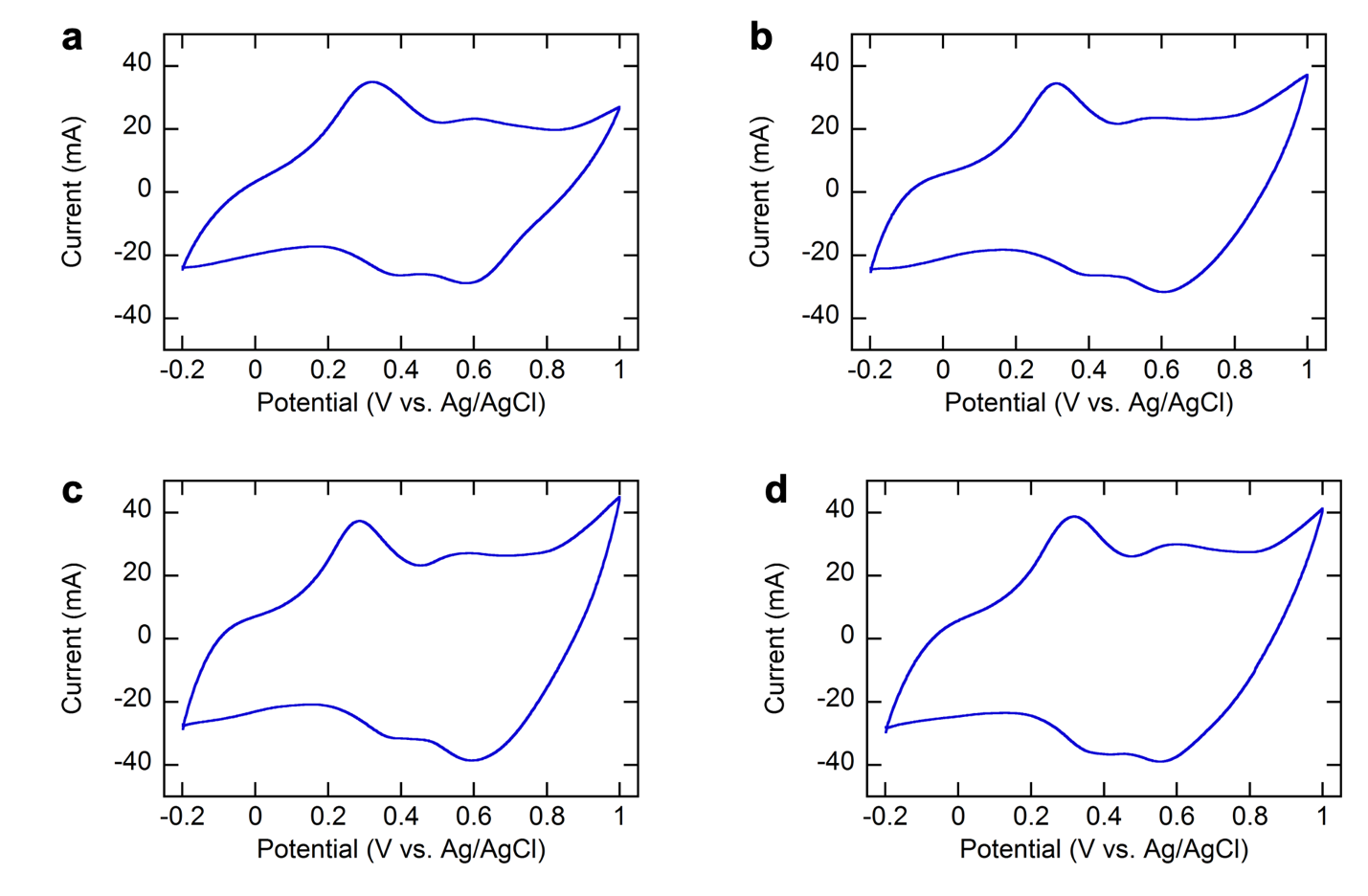


**Supplementary Figure 16.** Cyclic voltammograms of PANI/CNT and PANI-Co_3_O_4_/CNT electrodes with Co_3_O_4_ loadings varied. a) PANI/CNT electrode and b–d) PANI-Co_3_O_4_/CNT electrodes with the duration of Co(OH)_2_ deposition being (b) 1 min, (c) 2 min, and (d) 4 min. For (b–d), Co(OH)_2_ was electrodeposited in 0.1 M Co(NO_3_)_2_ solution at -1.0 V vs Ag/AgCl for various durations, then converted into Co_3_O_4_ by heat treatment at 200°C for 1 h. Finally, PANI was electropolymerized in 0.2 M aniline + 0.5 M H_2_SO_4_ at a constant current of 3 mA cm^-2^ for 5 min. For all cases, scan rates were 50 mV s^-1^.


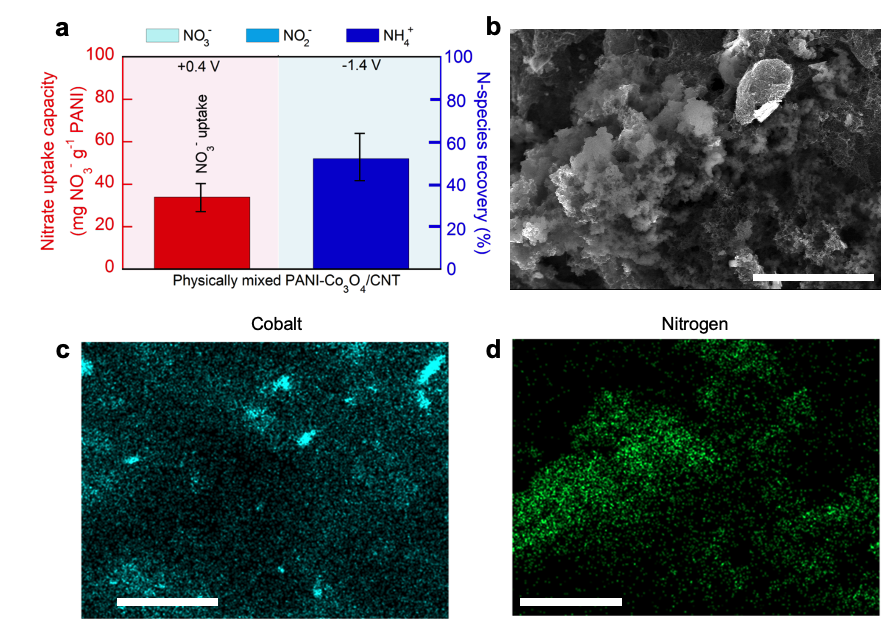


**Supplementary Figure 17.** a) The reactive separation performances of physically-mixed PANI-Co_3_O_4_/CNT. For this experiment, slurry of active materials containing 200 mg CNT, 100 mg PANI, and 100 mg Co_3_O_4_ in 20 mL DMF was prepared by physically mixing and sonicating for 2 h and then drop-casted on Ti mesh electrode. Error bars indicate the standard error of the mean (n = 2). b–d) Based on elemental analysis performed in SEM, N content turned out to be 3.47 wt%, which is within acceptable range considering the cotent of nitrogen in aniline. Scale bar is 10 μm.


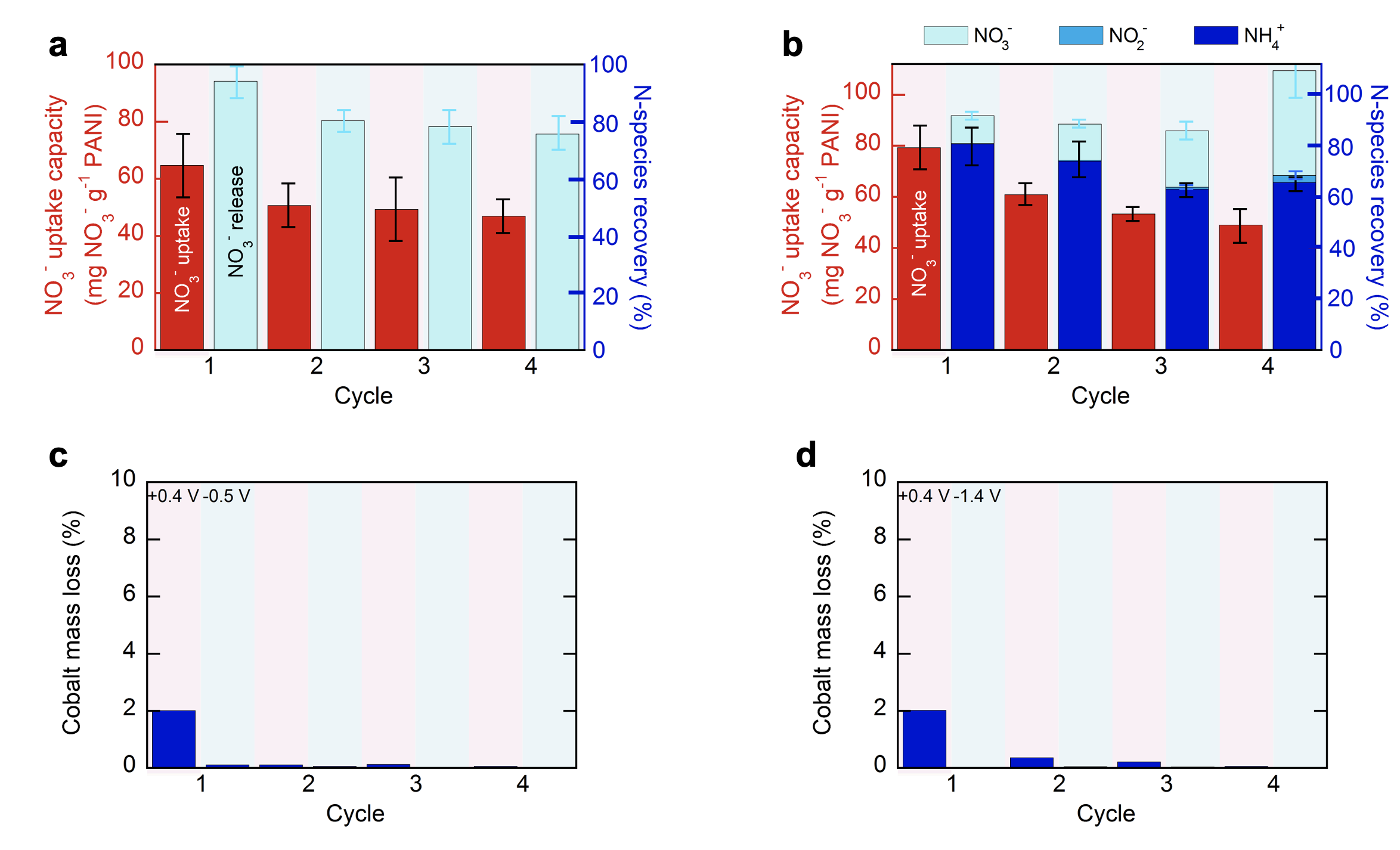


**Supplementary Figure 18.** a–b) Recyclability of PANI-Co_3_O_4_/CNT electrode over four cycles, as given by nitrate uptake capacity and nitrogen-species recovery; (a) adsorption: +0.4 V vs Ag/AgCl in 5 mM NaNO_3_ + 5 mM NaCl for 0.5 h, desorption: -0.5 V vs Ag/AgCl in 0.1 M NaCl for 1 h, (b) adsorption: +0.4 V vs Ag/AgCl in 5 mM NaNO_3_ + 5 mM NaCl for 0.5 h, desorption: -1.4 V vs Ag/AgCl in 0.1 M NaCl for 1 h. Error bars indicate the standard error of the mean (n = 2). c–d) Corresponding cobalt leaching during adsorption/desorption cycles measured by ICP analysis.


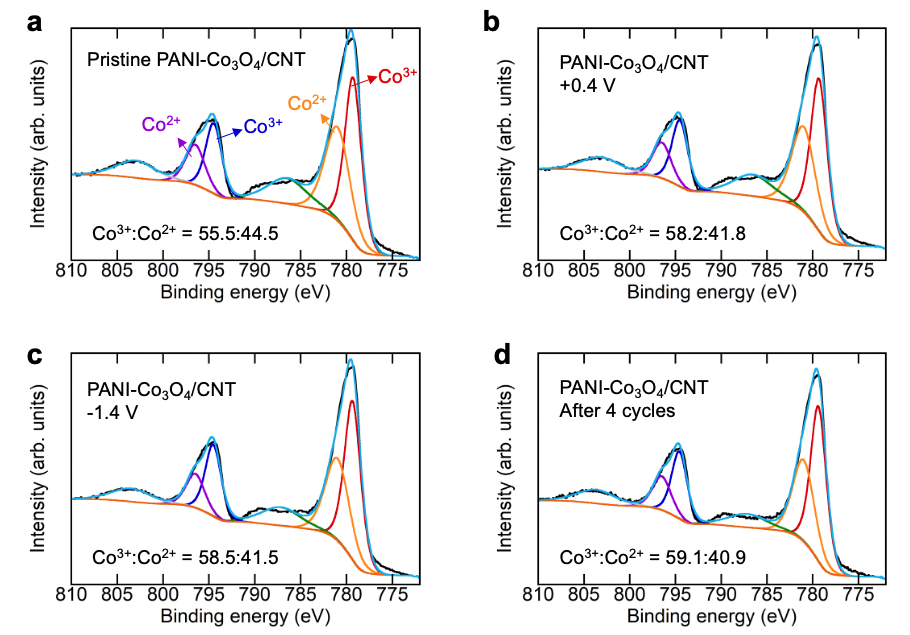


**Supplementary Figure 19.** High-resolution XPS spectra of Co*2p* for (a) pristine PANI-Co_3_O_4_/CNT, (b) PANI-Co_3_O_4_/CNT charged at +0.4 V vs Ag/AgCl in 5 mM NaNO_3_ + 5 mM NaCl for 0.5 h during electrosorption, (c) PANI-Co_3_O_4_/CNT charged at +0.4 V vs Ag/AgCl in 5 mM NaNO_3_ + 5 mM NaCl for electrosorption, followed by releasing at -1.4 V vs Ag/AgCl in 0.1 M NaCl for 1h during desorption, and (d) PANI-Co_3_O_4_/CNT after 4 successive cycles of adsorption and desorption at +0.4 V and -1.4 V vs Ag/AgCl (adsorption: +0.4 V vs Ag/AgCl in 5 mM NaNO_3_ + 5 mM NaCl for 0.5 h, desorption: -1.4 V vs Ag/AgCl in 0.1 M NaCl for 1h). Following the subtraction of a Shirley background from the region of interest, the spectra were fit into their components. Curves were fitted following published literature^2, 3^.


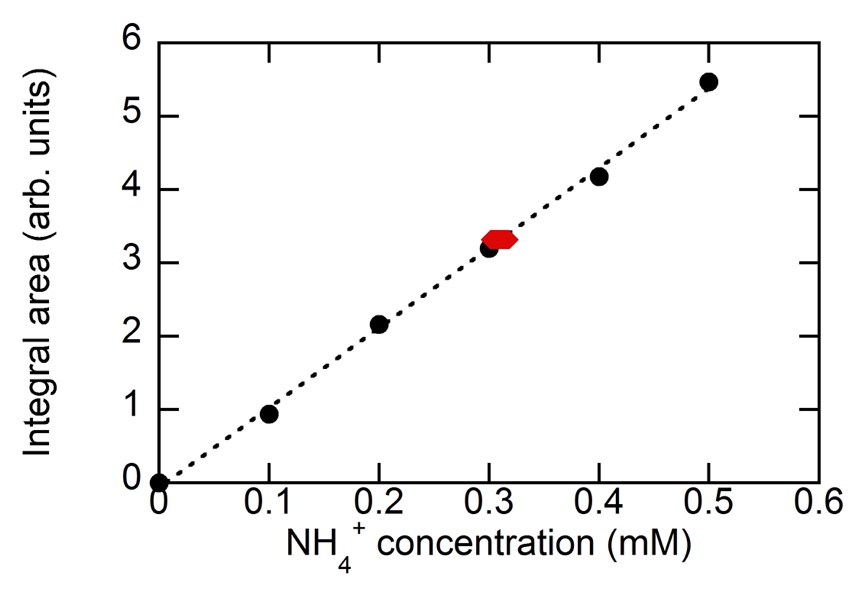


**Supplementary Figure 20.** A calibration curve based on ^1^H NMR signals. For standard solutions (0.1, 0.2, 0.3, 0.4, and 0.5 mM), black circles represent concentrations and NMR signal integrations, which result in a standard calibration line with a R^2^ value of 0.9984. The red hexagon indicates the concentration of ^15^NH_4_^+^ in the desorption electrolyte after the regeneration of the ^15^NO_3_^-^-adsorbing PANI-Co_3_O_4_/CNT electrode at -1.4 V vs Ag/AgCl. The estimated concentration of ^15^NH_4_^+^ was 0.31 mM, which was close to the estimated value (0.32 mM) determined by the colorimetric indophenol blue method, thus confirming the reliability of our method.


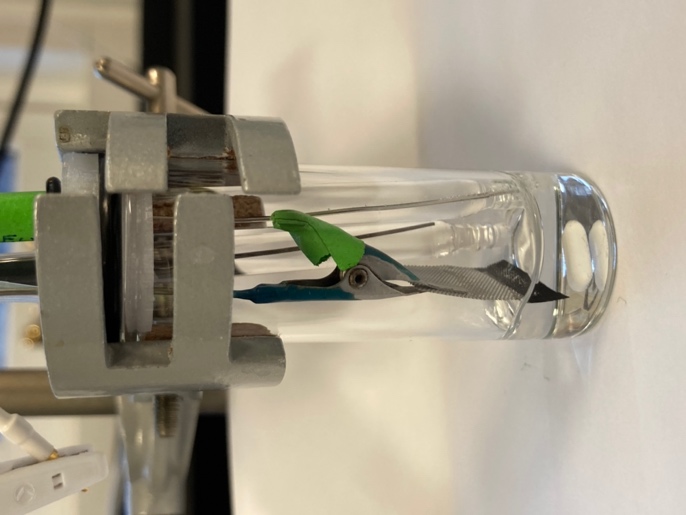


**Supplementary Figure 21.** A photographic picture of the VC-2 electrochemical cell used in this study.


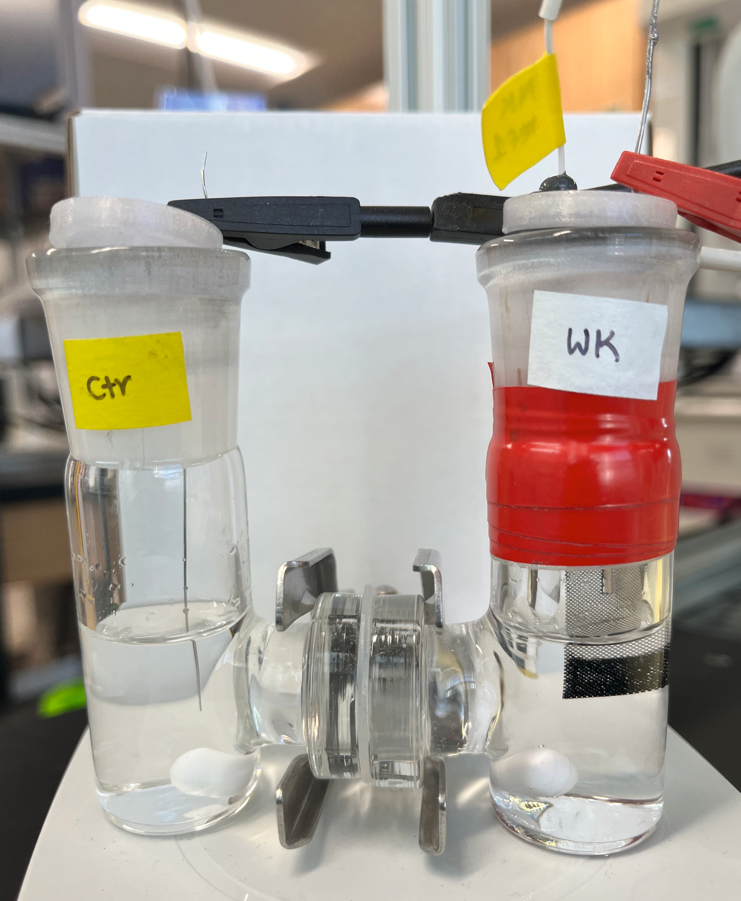


**Supplementary Figure 22.** A photographic picture of the H-type cell used in this study.


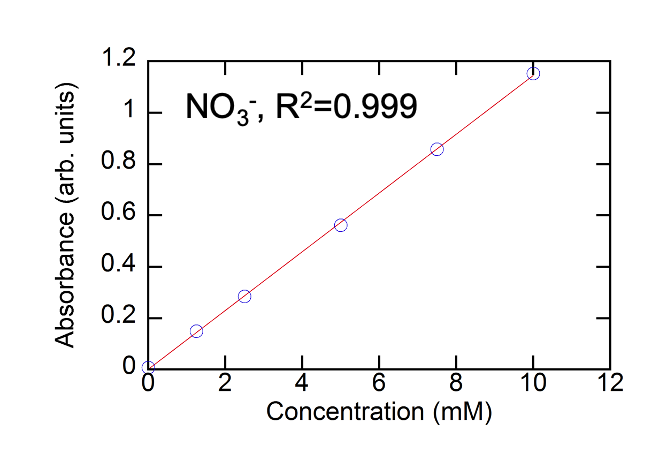


**Supplementary Figure 23.** A calibration curves for colorimetric nitrite assay using salicylic acid method.


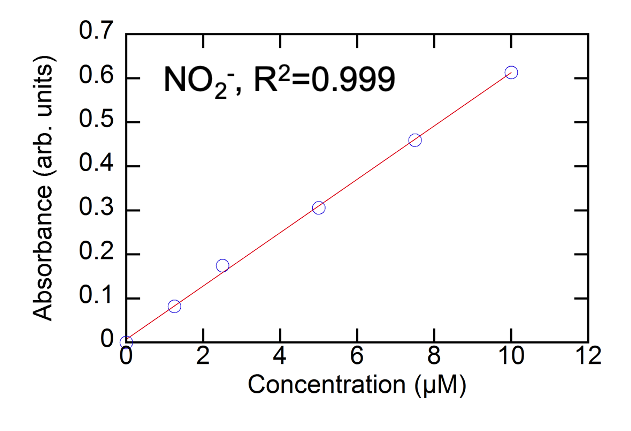


**Supplementary Figure 24.** A calibration curves for colorimetric nitrite assay using Griess reagent.


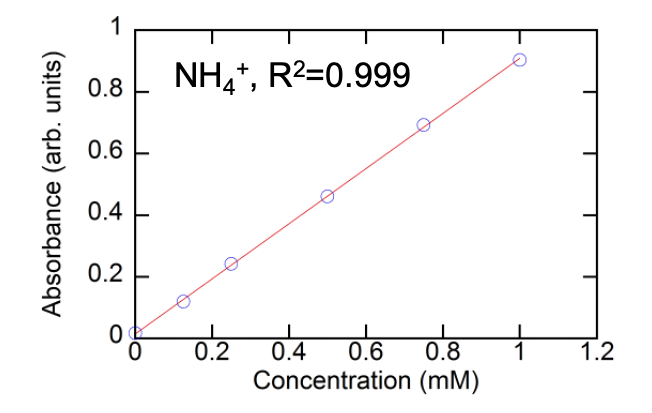


**Supplementary Figure 25.** A calibration curves for colorimetric ammonium assay using the indophenol blue method.

**Supplementary Table 1.** State-of-the art electrocatalysts for electrochemical nitrate reduction. The practical concentration levels of nitrate are also given.

| **Material** | **Starting nitrate concentration (mM)** | **Reference** |
| --- | --- | --- |
| Ru-dispersed Cu nanowire | 10 mM | 4 |
| Cu-PTCDA | 8.1 mM | 5 |
| Cu-Ni alloy | 1–100 mM | 6 |
| Ti | 10–1000 mM | 7 |
| Cu/Cu_2_O Nanowire array | 14.3 mM | 8 |
| (S) N-modified, carbon-supported Fe or Cu single-atom catalysts (SACs) | 7.1 mM | 9 |
| Fe single atom catalyst | 500 mM | 10 |
| Fe-PPy Single atom catalysts | 100 mM | 11 |
| Pd | 20 mM | 12 |
| Ru nanocluster | 1000 mM | 13 |
| Ti/RuO_2_ | 41.61 mM | 14 |
| CoP | 10–100 mM | 15 |
| Oxide-derived silver | 100 mM | 16 |
| **Source** | **Nitrate concentration (mM)** | **Reference** |
| Havana Lowlands (Illinois, US) - an individual well with the highest nitrate detected | 1.3–3.4 mM  (18–48 mg-N/L) | 17 |
| Marsh creek (Idaho, US) - 1^st^-ranked nitrate priority areas | 0.51–2.9 mM  (7.16–40 mg-N/L) | 18 |
| Upper Big Walnut Creek Watershed (Ohio, US) | <1.2 mM (<1.71 mg-N/L) | 19 |

**Supplementary Table 2.** Mass loading of PANI and Co_3_O_4_ in PANI-Co_3_O_4_/CNT composites with various duration of Co(OH)_2_ deposition. Co(OH)_2_ was electrodeposited in 0.1 M Co(NO_3_)_2_ solution at -1.0 V vs Ag/AgCl for 1, 2, and 4 min, then converted into Co_3_O_4_ by heat treatment at 200°C for 1 h. For all cases, PANI was electropolymerized in 0.2 M aniline + 0.5 M H_2_SO_4_ at a constant current of 3 mA cm^-2^ for 5 min.

| Duration of Co(OH)_2_ deposition | Mass of PANI | Mass of Co_3_O_4_ |
| --- | --- | --- |
| 0 min | 1.11 ± 0.04 mg | - |
| 1 min | 1.17 ± 0.08 mg | 0.32 ± 0.04 mg |
| 2 min | 1.09 ± 0.04 mg | 0.78 ± 0.11 mg |
| 4 min | 1.09 ± 0.07 mg | 1.08 ± 0.02 mg |

**Supplementary Table 3.** The composition of corn/soy tile drainage sample collected from University of Illinois Energy Farm.

| Element | Concentrations or value (mg/L) | Standard deviation |
| --- | --- | --- |
| B | 0.018 | 0.002 |
| Ba | 0.029 | 0.008 |
| Ca | 54.250 | 11.662 |
| Mg | 24.922 | 3.049 |
| Na | 3.146 | 0.496 |
| Si | 4.301 | 0.370 |
| Sr | 0.082 | 0.015 |
| Cl^-^ | 10.390 | 1.179 |
| NO_3_^-^ | 16.740 | 5.259 |
| SO_4_^2-^ | 44.590 | 4.877 |
| Conductivity | 505 µS/cm |  |
| pH | 7.6 |  |

**Supplementary Table 4.** Optimized structures presented in the Supplementary Fig. 13 (in Å).

PS-NO_3_

| C | 3.871522 | 15.065450 | 14.402300 |
| --- | --- | --- | --- |
| C | 3.803768 | 13.645525 | 14.235149 |
| C | 5.167536 | 15.673550 | 14.580099 |
| C | 4.952302 | 12.905499 | 14.264000 |
| C | 6.316050 | 14.934275 | 14.606474 |
| C | 6.248598 | 13.513801 | 14.443625 |
| C | 8.686867 | 12.823351 | 14.453276 |
| C | 9.393341 | 13.984799 | 14.061475 |
| C | 9.423486 | 11.660650 | 14.790350 |
| C | 10.783954 | 13.989800 | 14.034200 |
| C | 10.802487 | 11.662700 | 14.754801 |
| C | 11.511154 | 12.830925 | 14.387650 |
| C | 13.940731 | 13.530800 | 14.361199 |
| C | 13.860059 | 14.921000 | 14.701051 |
| C | 15.239562 | 12.966876 | 14.074800 |
| C | 14.997504 | 15.675426 | 14.737076 |
| C | 16.376244 | 13.721199 | 14.110400 |
| C | 16.296335 | 15.109725 | 14.454475 |
| C | 18.731083 | 15.787175 | 14.448075 |
| C | 19.446331 | 14.616699 | 14.790900 |
| C | 19.453979 | 16.952850 | 14.102075 |
| C | 0.714423 | 14.608450 | 14.769800 |
| C | 0.709694 | 16.941650 | 14.072825 |
| C | 1.433675 | 15.768001 | 14.396299 |
| H | 2.859703 | 13.151300 | 14.026475 |
| H | 5.214966 | 16.750824 | 14.751525 |
| H | 4.905637 | 11.829000 | 14.090175 |
| H | 7.260175 | 15.430326 | 14.811425 |
| H | 8.870831 | 14.868275 | 13.705800 |
| H | 8.895561 | 10.752850 | 15.079750 |
| H | 11.291695 | 14.875401 | 13.660026 |
| H | 11.354297 | 10.762475 | 15.027775 |
| H | 12.910198 | 15.371850 | 14.975825 |
| H | 15.297093 | 11.916500 | 13.791249 |
| H | 14.940718 | 16.726349 | 15.019449 |
| H | 17.325602 | 13.272076 | 13.832074 |
| H | 18.930361 | 13.729826 | 15.150351 |
| H | 18.912754 | 17.864176 | 13.842800 |
| H | 1.227134 | 13.714700 | 15.113676 |
| H | 1.246311 | 17.848675 | 13.797951 |
| H | 7.012020 | 11.659451 | 14.547550 |
| H | 13.183406 | 11.672875 | 14.159575 |
| H | 17.069155 | 16.962425 | 14.655575 |
| H | 3.113131 | 16.921000 | 14.331250 |
| N | 7.299274 | 12.666625 | 14.462725 |
| N | 12.899854 | 12.679926 | 14.308000 |
| N | 17.342464 | 15.953300 | 14.515776 |
| N | 2.822757 | 15.914824 | 14.391200 |
| N | 5.937256 | 9.248276 | 14.424125 |
| N | 13.744111 | 9.239301 | 14.578250 |
| N | 16.595423 | 19.491001 | 14.295925 |
| N | 4.269512 | 19.352449 | 14.412374 |
| O | 6.656167 | 10.035375 | 15.134775 |
| O | 5.823905 | 8.044724 | 14.763350 |
| O | 5.351580 | 9.695550 | 13.399776 |
| O | 13.705334 | 10.119100 | 13.643651 |
| O | 13.258444 | 9.516725 | 15.708850 |
| O | 14.260645 | 8.119525 | 14.345175 |
| O | 16.517124 | 18.525700 | 15.137650 |
| O | 15.939114 | 20.540625 | 14.504576 |
| O | 17.329485 | 19.370075 | 13.277250 |
| O | 3.446346 | 18.587599 | 13.798025 |
| O | 4.358999 | 20.558224 | 14.072925 |
| O | 4.981923 | 18.883749 | 15.343100 |

EB-NO_3_

| C | 3.799860 | 14.404551 | 14.003449 |
| --- | --- | --- | --- |
| C | 5.085580 | 14.942925 | 14.109950 |
| C | 6.236815 | 14.162324 | 14.004450 |
| C | 6.119388 | 12.765500 | 13.809400 |
| C | 4.807261 | 12.217900 | 13.705074 |
| C | 3.644785 | 13.022150 | 13.780875 |
| C | 8.453064 | 11.946825 | 13.747001 |
| C | 9.261553 | 13.029675 | 13.183500 |
| C | 10.628218 | 12.955125 | 13.110150 |
| C | 11.363677 | 11.810676 | 13.644976 |
| C | 10.558305 | 10.729250 | 14.191900 |
| C | 9.201364 | 10.781950 | 14.203074 |
| C | 13.853592 | 12.203225 | 13.500725 |
| C | 14.168810 | 13.523325 | 13.962725 |
| C | 15.464565 | 13.992375 | 13.907576 |
| C | 16.550730 | 13.171875 | 13.465450 |
| C | 16.253691 | 11.855200 | 13.014700 |
| C | 14.950623 | 11.407350 | 13.007750 |
| C | 19.086933 | 13.335700 | 13.459425 |
| C | 20.096619 | 14.334075 | 13.232400 |
| C | 0.662161 | 14.052850 | 13.286474 |
| C | 1.117058 | 12.733049 | 13.605274 |
| C | 0.099892 | 11.725650 | 13.805125 |
| C | 19.533352 | 12.004450 | 13.733850 |
| H | 5.185992 | 16.007849 | 14.318325 |
| H | 7.211764 | 14.620975 | 14.154050 |
| H | 4.707410 | 11.139925 | 13.566375 |
| H | 2.945788 | 15.054150 | 14.166599 |
| H | 8.763129 | 13.904400 | 12.767200 |
| H | 11.193312 | 13.759525 | 12.639225 |
| H | 11.088329 | 9.853951 | 14.566125 |
| H | 8.616220 | 9.946450 | 14.586151 |
| H | 13.378397 | 14.153349 | 14.368425 |
| H | 15.687328 | 15.005125 | 14.249675 |
| H | 17.038971 | 11.212225 | 12.623175 |
| H | 14.723599 | 10.410775 | 12.631200 |
| H | 19.776934 | 15.346900 | 12.987775 |
| H | 1.366706 | 14.845125 | 13.055050 |
| H | 0.420055 | 10.714900 | 14.058000 |
| H | 18.818649 | 11.213225 | 13.948925 |
| H | 17.718544 | 14.832300 | 13.357300 |
| H | 2.515573 | 11.301250 | 13.916000 |
| N | 7.141519 | 11.831075 | 13.879199 |
| N | 12.660763 | 11.595625 | 13.610975 |
| N | 17.795103 | 13.772050 | 13.451324 |
| N | 2.409468 | 12.336801 | 13.730325 |
| N | 3.107012 | 8.844126 | 13.753475 |
| N | 17.297220 | 17.263575 | 13.622725 |
| O | 2.549999 | 9.701525 | 14.538024 |
| O | 3.117421 | 7.629700 | 14.079150 |
| O | 3.634023 | 9.243800 | 12.675900 |
| O | 17.622620 | 16.382175 | 12.736199 |
| O | 17.019463 | 16.880125 | 14.794300 |
| O | 17.263168 | 18.475651 | 13.296725 |

ES-NO_3_

| C | 3.779935 | 14.371750 | 14.152925 |
| --- | --- | --- | --- |
| C | 5.062186 | 14.916150 | 14.243899 |
| C | 6.212091 | 14.142525 | 14.072426 |
| C | 6.079476 | 12.769750 | 13.780925 |
| C | 4.785986 | 12.220900 | 13.650499 |
| C | 3.631800 | 13.001699 | 13.845900 |
| C | 8.526757 | 11.935900 | 13.625351 |
| C | 9.348564 | 13.085225 | 13.469049 |
| C | 10.738644 | 12.985399 | 13.430526 |
| C | 11.383519 | 11.729050 | 13.537049 |
| C | 10.556539 | 10.578200 | 13.665999 |
| C | 9.188628 | 10.676975 | 13.709325 |
| C | 13.934806 | 12.126775 | 13.476101 |
| C | 14.129480 | 13.528501 | 13.613375 |
| C | 15.405727 | 14.051725 | 13.602275 |
| C | 16.570341 | 13.229425 | 13.468025 |
| C | 16.370640 | 11.829000 | 13.308925 |
| C | 15.098716 | 11.308775 | 13.315800 |
| C | 19.090797 | 13.427875 | 13.550501 |
| C | 20.111038 | 14.364725 | 13.212276 |
| C | 0.678325 | 14.048224 | 13.279825 |
| C | 1.098089 | 12.761700 | 13.717050 |
| C | 0.071761 | 11.824500 | 14.053925 |
| C | 19.507216 | 12.140075 | 13.982124 |
| H | 5.170389 | 15.969925 | 14.497126 |
| H | 7.186084 | 14.599951 | 14.220975 |
| H | 4.668018 | 11.156150 | 13.431326 |
| H | 2.918654 | 14.994550 | 14.381351 |
| H | 6.820837 | 10.905625 | 13.656124 |
| H | 8.909332 | 14.069975 | 13.335800 |
| H | 11.309451 | 13.897200 | 13.273475 |
| H | 11.018998 | 9.593800 | 13.741199 |
| H | 8.594967 | 9.769525 | 13.818675 |
| H | 12.897820 | 10.440750 | 13.449325 |
| H | 13.289162 | 14.202525 | 13.754874 |
| H | 15.544803 | 15.125825 | 13.741800 |
| H | 17.205910 | 11.159201 | 13.124225 |
| H | 14.969133 | 10.236675 | 13.168751 |
| H | 19.817511 | 15.353675 | 12.860075 |
| H | 1.405329 | 14.789200 | 12.956551 |
| H | 0.363357 | 10.837800 | 14.412950 |
| H | 18.785095 | 11.399775 | 14.316000 |
| H | 17.704603 | 14.909400 | 13.330475 |
| H | 2.485697 | 11.302825 | 13.973451 |
| N | 7.148790 | 11.870475 | 13.672425 |
| N | 12.743805 | 11.450100 | 13.485201 |
| N | 17.783491 | 13.867000 | 13.464725 |
| N | 2.396026 | 12.332650 | 13.834275 |
| N | 3.112351 | 8.660650 | 13.802876 |
| N | 17.136225 | 17.452974 | 13.460525 |
| O | 2.377182 | 9.550301 | 14.366250 |
| O | 2.825327 | 7.441075 | 13.948600 |
| O | 4.112294 | 9.017475 | 13.113350 |
| O | 17.737907 | 16.548624 | 12.769200 |
| O | 16.442568 | 17.112101 | 14.461425 |
| O | 17.248959 | 18.660351 | 13.121225 |

LB-NO_3_

| C | 2.748817 | 12.675526 | 13.498375 |
| --- | --- | --- | --- |
| C | 3.814433 | 12.894575 | 14.419225 |
| C | 3.100481 | 12.263374 | 12.187925 |
| C | 5.144020 | 12.750051 | 14.065724 |
| C | 4.430237 | 12.104775 | 11.838500 |
| C | 5.498886 | 12.356050 | 12.749375 |
| C | 8.049943 | 12.407700 | 12.741976 |
| C | 8.383473 | 13.312849 | 13.782425 |
| C | 9.131422 | 11.765600 | 12.070150 |
| C | 9.707461 | 13.553474 | 14.103050 |
| C | 10.455601 | 12.007150 | 12.390900 |
| C | 10.788284 | 12.926149 | 13.417999 |
| C | 13.353400 | 13.064125 | 13.368026 |
| C | 13.708097 | 12.552400 | 12.094925 |
| C | 14.416939 | 13.481874 | 14.219425 |
| C | 15.038872 | 12.455850 | 11.725825 |
| C | 15.748053 | 13.378175 | 13.853450 |
| C | 16.101900 | 12.845850 | 12.588800 |
| C | 18.658791 | 12.805525 | 12.615526 |
| C | 18.991344 | 12.746850 | 13.991974 |
| C | 19.735729 | 12.894000 | 11.687450 |
| C | 20.315546 | 12.759900 | 14.391400 |
| C | 21.060396 | 12.907926 | 12.086175 |
| C | 0.185863 | 12.823775 | 13.461425 |
| H | 3.574067 | 13.171500 | 15.446099 |
| H | 2.340975 | 12.023050 | 11.449349 |
| H | 5.901151 | 12.893476 | 14.831875 |
| H | 4.672428 | 11.765825 | 10.830750 |
| H | 6.784615 | 11.599400 | 11.370925 |
| H | 7.614573 | 13.867400 | 14.313624 |
| H | 8.900853 | 11.036150 | 11.293675 |
| H | 9.931265 | 14.271826 | 14.891701 |
| H | 11.222360 | 11.445200 | 11.865900 |
| H | 12.053378 | 13.793950 | 14.712601 |
| H | 12.951581 | 12.261400 | 11.372425 |
| H | 14.175703 | 13.918249 | 15.188874 |
| H | 15.278539 | 12.071575 | 10.734150 |
| H | 16.506922 | 13.764050 | 14.528450 |
| H | 17.394247 | 12.579375 | 11.075125 |
| H | 18.220896 | 12.648000 | 14.752049 |
| H | 19.508596 | 12.979751 | 10.625150 |
| H | 20.542042 | 12.691601 | 15.455299 |
| H | 0.620766 | 13.028174 | 11.327000 |
| H | 1.457511 | 12.950450 | 15.009125 |
| N | 6.778465 | 12.121550 | 12.281475 |
| N | 6.884970 | 9.549126 | 9.924675 |
| N | 12.065022 | 13.256825 | 13.845051 |
| N | 17.385805 | 12.730350 | 12.083800 |
| N | 17.904713 | 16.466625 | 11.889825 |
| N | 1.464551 | 12.827250 | 13.995775 |
| O | 7.179966 | 9.083700 | 11.062900 |
| O | 6.712934 | 10.818450 | 9.789775 |
| O | 6.759527 | 8.798325 | 8.920725 |
| O | 18.002289 | 15.959724 | 13.049525 |
| O | 17.599430 | 15.736850 | 10.896550 |
| O | 18.112652 | 17.711500 | 11.722425 |

LS-NO_3_

| C | 2.737005 | 12.390075 | 13.651601 |
| --- | --- | --- | --- |
| C | 3.645621 | 11.890550 | 14.623301 |
| C | 3.278476 | 12.725350 | 12.390250 |
| C | 4.983818 | 11.651175 | 14.332025 |
| C | 4.628571 | 12.478675 | 12.097850 |
| C | 5.483108 | 11.920625 | 13.051701 |
| C | 8.129174 | 12.002950 | 12.873350 |
| C | 8.315886 | 13.249000 | 13.464776 |
| C | 9.242783 | 11.261675 | 12.463700 |
| C | 9.614933 | 13.732275 | 13.648075 |
| C | 10.543039 | 11.740849 | 12.651150 |
| C | 10.763344 | 12.994901 | 13.258700 |
| C | 13.337666 | 13.327024 | 13.179726 |
| C | 13.793182 | 12.418850 | 12.199800 |
| C | 14.322310 | 14.135376 | 13.809750 |
| C | 15.146479 | 12.377000 | 11.836475 |
| C | 15.662841 | 14.102099 | 13.436524 |
| C | 16.086292 | 13.223425 | 12.431825 |
| C | 18.736515 | 13.059075 | 12.431225 |
| C | 18.927214 | 12.800776 | 13.786276 |
| C | 19.847855 | 13.126124 | 11.585975 |
| C | 20.223547 | 12.621249 | 14.274449 |
| C | 21.146944 | 12.942875 | 12.071475 |
| C | 0.162980 | 12.694751 | 13.441075 |
| H | 3.275740 | 11.644975 | 15.619600 |
| H | 2.666002 | 13.169100 | 11.611300 |
| H | 5.626365 | 11.212025 | 15.095276 |
| H | 4.995715 | 12.701075 | 11.096175 |
| H | 6.855088 | 10.428775 | 13.068625 |
| H | 6.735392 | 11.136476 | 11.578650 |
| H | 7.464911 | 13.847526 | 13.790150 |
| H | 9.103513 | 10.281025 | 12.006175 |
| H | 9.745763 | 14.709001 | 14.116125 |
| H | 11.371088 | 11.104425 | 12.351425 |
| H | 11.925263 | 14.369576 | 14.165150 |
| H | 13.103788 | 11.773274 | 11.662900 |
| H | 14.014546 | 14.840401 | 14.582750 |
| H | 15.449513 | 11.697575 | 11.039075 |
| H | 16.371321 | 14.791475 | 13.895875 |
| H | 17.390259 | 12.872225 | 10.908299 |
| H | 17.492649 | 14.441250 | 11.516700 |
| H | 18.081667 | 12.740424 | 14.470776 |
| H | 19.714458 | 13.334126 | 10.523849 |
| H | 20.354164 | 12.420176 | 15.338426 |
| H | 0.759188 | 12.995650 | 11.361526 |
| H | 1.298072 | 12.281125 | 15.049174 |
| N | 6.796639 | 11.364025 | 12.641425 |
| N | 6.775666 | 9.397650 | 10.021550 |
| N | 12.011196 | 13.556775 | 13.560100 |
| N | 17.419760 | 13.380450 | 11.801325 |
| N | 18.015203 | 16.694174 | 11.981125 |
| N | 1.404428 | 12.502700 | 14.062699 |
| O | 7.043750 | 8.780650 | 11.090400 |
| O | 6.649523 | 10.687450 | 10.054900 |
| O | 6.628315 | 8.799750 | 8.929424 |
| O | 18.258453 | 16.202675 | 13.114349 |
| O | 17.602695 | 15.913425 | 11.028925 |
| O | 18.156425 | 17.917200 | 11.738325 |

**Supplementary Table 5.** Optimized structures presented in the Supplementary Fig. 14 (in Å).

PS-Cl

| C | 3.837725 | 14.587675 | 13.815574 |
| --- | --- | --- | --- |
| C | 3.706397 | 13.179575 | 13.545901 |
| C | 5.158071 | 15.128475 | 14.081550 |
| C | 4.808536 | 12.384825 | 13.573900 |
| C | 6.261616 | 14.334925 | 14.102075 |
| C | 6.128467 | 12.925351 | 13.841100 |
| C | 8.528332 | 12.201200 | 13.872500 |
| C | 9.229837 | 13.307200 | 13.346125 |
| C | 9.247390 | 11.085175 | 14.354575 |
| C | 10.620636 | 13.300550 | 13.313749 |
| C | 10.629858 | 11.077650 | 14.320725 |
| C | 11.334628 | 12.188050 | 13.807151 |
| C | 13.732712 | 12.903351 | 13.738175 |
| C | 13.598456 | 14.301950 | 14.055499 |
| C | 15.052267 | 12.375150 | 13.442776 |
| C | 14.699842 | 15.097874 | 14.057900 |
| C | 16.154406 | 13.170375 | 13.450450 |
| C | 16.020090 | 14.568725 | 13.767475 |
| C | 18.419736 | 15.292674 | 13.741250 |
| C | 19.127495 | 14.180975 | 14.244850 |
| C | 19.131255 | 16.414700 | 13.262776 |
| C | 0.728341 | 14.186725 | 14.256351 |
| C | 0.723828 | 16.419201 | 13.271825 |
| C | 1.436475 | 15.302850 | 13.761750 |
| H | 2.742375 | 12.758575 | 13.273175 |
| H | 5.235194 | 16.193025 | 14.306475 |
| H | 4.730483 | 11.320275 | 13.351075 |
| H | 7.226786 | 14.757600 | 14.369050 |
| H | 8.700566 | 14.138100 | 12.886526 |
| H | 8.703653 | 10.224375 | 14.742175 |
| H | 11.137755 | 14.126050 | 12.830025 |
| H | 11.183847 | 10.212125 | 14.682201 |
| H | 12.632315 | 14.710476 | 14.340974 |
| H | 15.129904 | 11.320150 | 13.177875 |
| H | 14.621294 | 16.153350 | 14.319874 |
| H | 17.121239 | 12.761275 | 13.168500 |
| H | 18.603271 | 13.346350 | 14.703575 |
| H | 18.582036 | 17.280950 | 12.895849 |
| H | 1.250091 | 13.356775 | 14.726450 |
| H | 1.272711 | 17.288400 | 12.911600 |
| H | 6.824490 | 11.011150 | 13.823025 |
| H | 13.029426 | 10.989575 | 13.642424 |
| H | 16.719041 | 16.480824 | 13.867725 |
| H | 3.132698 | 16.498725 | 13.874675 |
| N | 7.134108 | 12.050375 | 13.840200 |
| N | 12.726872 | 12.030350 | 13.713725 |
| N | 17.025414 | 15.441975 | 13.798624 |
| N | 2.829590 | 15.459201 | 13.825674 |
| Cl | 6.273866 | 9.168875 | 13.885325 |
| Cl | 13.561447 | 9.152600 | 13.604800 |
| Cl | 16.189593 | 18.324275 | 13.907550 |
| Cl | 3.666579 | 18.345575 | 13.894451 |

EB-Cl

| C | 3.782676 | 13.785350 | 12.969100 |
| --- | --- | --- | --- |
| C | 5.083629 | 14.283200 | 13.083975 |
| C | 6.208443 | 13.459775 | 13.029000 |
| C | 6.040697 | 12.062800 | 12.875700 |
| C | 4.718922 | 11.557225 | 12.760951 |
| C | 3.587188 | 12.401750 | 12.787101 |
| C | 8.330208 | 11.182675 | 12.842850 |
| C | 9.125080 | 12.234800 | 12.212725 |
| C | 10.487782 | 12.157450 | 12.137225 |
| C | 11.215704 | 11.034625 | 12.718800 |
| C | 10.424497 | 9.974175 | 13.317950 |
| C | 9.069778 | 10.033800 | 13.347276 |
| C | 13.655761 | 11.519875 | 12.537600 |
| C | 13.884970 | 12.833375 | 13.061026 |
| C | 15.144995 | 13.387850 | 13.021401 |
| C | 16.265304 | 12.658074 | 12.515600 |
| C | 16.042729 | 11.364725 | 11.963675 |
| C | 14.777094 | 10.828525 | 11.960700 |
| C | 18.765495 | 12.849350 | 12.517650 |
| C | 19.786340 | 13.828000 | 12.271075 |
| C | 0.645430 | 13.508350 | 12.292625 |
| C | 1.065111 | 12.176450 | 12.611075 |
| C | 0.034438 | 11.190375 | 12.828299 |
| C | 19.176187 | 11.504450 | 12.778850 |
| H | 5.217101 | 15.351249 | 13.256525 |
| H | 7.197184 | 13.887174 | 13.183300 |
| H | 4.579841 | 10.477575 | 12.682401 |
| H | 2.949764 | 14.469925 | 13.089600 |
| H | 8.616151 | 13.076299 | 11.744275 |
| H | 11.055574 | 12.930176 | 11.618575 |
| H | 10.963338 | 9.120875 | 13.726924 |
| H | 8.482721 | 9.225900 | 13.783924 |
| H | 13.058692 | 13.380951 | 13.512775 |
| H | 15.313478 | 14.393026 | 13.410150 |
| H | 16.858034 | 10.816325 | 11.496850 |
| H | 14.600032 | 9.847900 | 11.522000 |
| H | 19.475439 | 14.846350 | 12.033300 |
| H | 1.367761 | 14.273800 | 12.029350 |
| H | 0.348533 | 10.176925 | 13.085376 |
| H | 18.441409 | 10.739475 | 13.019276 |
| H | 17.397529 | 14.370200 | 12.606224 |
| H | 2.428838 | 10.697100 | 12.881476 |
| N | 7.021966 | 11.092850 | 12.993824 |
| N | 12.511907 | 10.831175 | 12.671950 |
| N | 17.482109 | 13.296600 | 12.546250 |
| N | 2.342867 | 11.746325 | 12.731650 |
| Cl | 2.599020 | 8.707900 | 13.251075 |
| Cl | 17.221206 | 16.310000 | 12.639526 |

ES-Cl

| C | 3.765826 | 13.770100 | 13.068150 |
| --- | --- | --- | --- |
| C | 5.070137 | 14.260276 | 13.148449 |
| C | 6.184447 | 13.428526 | 13.018700 |
| C | 5.991539 | 12.054100 | 12.770750 |
| C | 4.679877 | 11.561075 | 12.641150 |
| C | 3.561821 | 12.396500 | 12.811325 |
| C | 8.393494 | 11.147800 | 12.691875 |
| C | 9.204765 | 12.259350 | 12.345550 |
| C | 10.592099 | 12.154475 | 12.319475 |
| C | 11.232902 | 10.931325 | 12.624276 |
| C | 10.418252 | 9.811975 | 12.938900 |
| C | 9.049468 | 9.917750 | 12.976025 |
| C | 13.737392 | 11.419350 | 12.536825 |
| C | 13.832761 | 12.811725 | 12.809651 |
| C | 15.064081 | 13.431650 | 12.804350 |
| C | 16.268785 | 12.710550 | 12.537549 |
| C | 16.163056 | 11.326375 | 12.224425 |
| C | 14.938431 | 10.707725 | 12.233250 |
| C | 18.750078 | 12.961650 | 12.626225 |
| C | 19.773401 | 13.874824 | 12.244050 |
| C | 0.634886 | 13.525675 | 12.280075 |
| C | 1.029219 | 12.237050 | 12.735350 |
| C | 20.472759 | 11.328075 | 13.127825 |
| C | 19.142405 | 11.674700 | 13.083425 |
| H | 5.224267 | 15.316300 | 13.366801 |
| H | 7.177611 | 13.840701 | 13.173699 |
| H | 4.512808 | 10.495675 | 12.471175 |
| H | 2.930538 | 14.435625 | 13.270675 |
| H | 6.661927 | 10.154025 | 12.820999 |
| H | 8.755438 | 13.199325 | 12.034550 |
| H | 11.169636 | 13.015200 | 11.989325 |
| H | 10.887870 | 8.855325 | 13.167199 |
| H | 8.451948 | 9.044650 | 13.238025 |
| H | 12.787040 | 9.668525 | 12.534399 |
| H | 12.950076 | 13.388126 | 13.076375 |
| H | 15.138873 | 14.493351 | 13.044275 |
| H | 17.038166 | 10.758800 | 11.920050 |
| H | 14.872299 | 9.653375 | 11.965200 |
| H | 19.480353 | 14.861400 | 11.882575 |
| H | 1.373166 | 14.236251 | 11.916050 |
| H | 0.293334 | 10.347650 | 13.505176 |
| H | 18.405680 | 10.972975 | 13.466100 |
| H | 17.339444 | 14.460900 | 12.529226 |
| H | 2.372493 | 10.730675 | 12.978150 |
| N | 7.017216 | 11.105125 | 12.737250 |
| N | 12.594110 | 10.670900 | 12.562650 |
| N | 17.446729 | 13.402575 | 12.565225 |
| N | 2.311213 | 11.770925 | 12.829199 |
| Cl | 2.513028 | 8.670000 | 13.219351 |
| Cl | 17.115026 | 16.454700 | 12.452800 |

LB-Cl

| C | 2.830780 | 12.339525 | 13.258851 |
| --- | --- | --- | --- |
| C | 3.916448 | 12.262400 | 14.174224 |
| C | 3.135170 | 12.391925 | 11.874450 |
| C | 5.230567 | 12.281950 | 13.753875 |
| C | 4.450187 | 12.388225 | 11.452400 |
| C | 5.539683 | 12.361025 | 12.370450 |
| C | 8.059548 | 12.496075 | 12.372425 |
| C | 8.336402 | 13.045774 | 13.650849 |
| C | 9.168426 | 12.107675 | 11.566700 |
| C | 9.637662 | 13.154425 | 14.096326 |
| C | 10.471965 | 12.228625 | 12.007150 |
| C | 10.742796 | 12.750649 | 13.297226 |
| C | 13.274016 | 12.909000 | 13.344200 |
| C | 13.593293 | 13.125500 | 11.979725 |
| C | 14.348205 | 12.820275 | 14.272299 |
| C | 14.912242 | 13.231499 | 11.585525 |
| C | 15.667070 | 12.902351 | 13.874125 |
| C | 15.990885 | 13.100076 | 12.506676 |
| C | 18.515414 | 12.985051 | 12.486575 |
| C | 18.801968 | 12.208175 | 13.639475 |
| C | 19.619670 | 13.503650 | 11.748650 |
| C | 20.108351 | 11.996150 | 14.029275 |
| C | 0.018044 | 13.277826 | 12.129675 |
| C | 0.299665 | 12.515800 | 13.291550 |
| H | 3.703074 | 12.177600 | 15.239701 |
| H | 2.344859 | 12.377175 | 11.127875 |
| H | 6.012932 | 12.180100 | 14.499825 |
| H | 4.674497 | 12.383550 | 10.384400 |
| H | 6.816395 | 12.111150 | 10.797125 |
| H | 7.539589 | 13.435949 | 14.277525 |
| H | 8.967952 | 11.676125 | 10.584475 |
| H | 9.827116 | 13.597950 | 15.073524 |
| H | 11.275992 | 11.862275 | 11.373050 |
| H | 11.971418 | 12.975775 | 14.877824 |
| H | 12.810927 | 13.265775 | 11.238000 |
| H | 14.121575 | 12.699375 | 15.331475 |
| H | 15.146859 | 13.439950 | 10.540550 |
| H | 16.440989 | 12.873225 | 14.635424 |
| H | 17.269061 | 13.714024 | 11.043250 |
| H | 18.008312 | 11.731425 | 14.207150 |
| H | 19.411419 | 14.116925 | 10.870325 |
| H | 20.303827 | 11.383575 | 14.909349 |
| H | 0.819165 | 13.740049 | 11.557450 |
| H | 1.551182 | 11.957400 | 14.765775 |
| N | 6.805774 | 12.336175 | 11.829000 |
| N | 11.996864 | 12.865575 | 13.863476 |
| N | 17.260341 | 13.253126 | 11.993875 |
| N | 1.560570 | 12.270875 | 13.794425 |
| Cl | 6.833226 | 11.565550 | 8.803150 |
| Cl | 17.261889 | 14.824025 | 9.305425 |

LS-Cl

| C | 2.807634 | 12.025100 | 13.301125 |
| --- | --- | --- | --- |
| C | 3.695254 | 11.137050 | 13.963275 |
| C | 3.356155 | 12.851600 | 12.296225 |
| C | 5.027187 | 11.013150 | 13.585275 |
| C | 4.696702 | 12.717350 | 11.911450 |
| C | 5.529960 | 11.786150 | 12.532425 |
| C | 8.143163 | 11.953800 | 12.461350 |
| C | 8.265375 | 13.077000 | 13.274950 |
| C | 9.290513 | 11.286400 | 12.022675 |
| C | 9.537718 | 13.526399 | 13.637125 |
| C | 10.562144 | 11.730800 | 12.388250 |
| C | 10.716389 | 12.874025 | 13.198975 |
| C | 13.254613 | 13.261250 | 13.184351 |
| C | 13.639941 | 12.706925 | 11.946025 |
| C | 14.280482 | 13.815700 | 13.993350 |
| C | 14.974884 | 12.750775 | 11.530625 |
| C | 15.605222 | 13.874801 | 13.568375 |
| C | 15.961027 | 13.341650 | 12.324075 |
| C | 18.577094 | 13.148201 | 12.281200 |
| C | 18.720779 | 12.476025 | 13.493225 |
| C | 19.713320 | 13.457200 | 11.528275 |
| C | 19.998412 | 12.128200 | 13.936675 |
| C | 0.082172 | 13.100700 | 11.963900 |
| C | 0.258266 | 12.428850 | 13.191350 |
| H | 3.315279 | 10.504850 | 14.766575 |
| H | 2.758394 | 13.609751 | 11.799375 |
| H | 5.661330 | 10.281625 | 14.085600 |
| H | 5.077849 | 13.326874 | 11.092225 |
| H | 6.907683 | 10.429525 | 11.913600 |
| H | 6.811753 | 11.738350 | 10.872625 |
| H | 7.380891 | 13.607626 | 13.628550 |
| H | 9.202361 | 10.399200 | 11.394975 |
| H | 9.622941 | 14.405125 | 14.277475 |
| H | 11.424005 | 11.152050 | 12.066850 |
| H | 11.850858 | 14.014176 | 14.433275 |
| H | 12.904244 | 12.288675 | 11.264550 |
| H | 14.020732 | 14.246100 | 14.960726 |
| H | 15.229156 | 12.351900 | 10.548200 |
| H | 16.349700 | 14.359550 | 14.199500 |
| H | 17.243343 | 13.301675 | 10.744300 |
| H | 17.358759 | 14.722025 | 11.656875 |
| H | 17.853734 | 12.210175 | 14.097500 |
| H | 19.611285 | 13.980825 | 10.577700 |
| H | 20.094885 | 11.596450 | 14.883851 |
| H | 0.923124 | 13.340876 | 11.321600 |
| H | 1.352444 | 11.440925 | 14.567150 |
| N | 6.844517 | 11.455925 | 11.936600 |
| N | 11.943694 | 13.369425 | 13.653199 |
| N | 17.279598 | 13.614926 | 11.722550 |
| N | 1.477688 | 12.005575 | 13.730749 |
| Cl | 6.873016 | 12.139200 | 9.033400 |
| Cl | 17.487578 | 16.567025 | 11.599900 |

**References**

1. Bednarczyk K, Matysiak W, Tański T, Janeczek H, Schab-Balcerzak E, Libera M. Effect of polyaniline content and protonating dopants on electroconductive composites. *Scientific Reports* **11**, 7487 (2021).

2. Wang Z, Wang W, Zhang L, Jiang D. Surface oxygen vacancies on Co3O4 mediated catalytic formaldehyde oxidation at room temperature. *Catalysis Science & Technology* **6**, 3845-3853 (2016).

3. Alex C, Sarma SC, Peter SC, John NS. Competing Effect of Co3+ Reducibility and Oxygen-Deficient Defects Toward High Oxygen Evolution Activity in Co3O4 Systems in Alkaline Medium. *ACS Applied Energy Materials* **3**, 5439-5447 (2020).

4. Chen F-Y*, et al.* Efficient conversion of low-concentration nitrate sources into ammonia on a Ru-dispersed Cu nanowire electrocatalyst. *Nature Nanotechnology*, (2022).

5. Chen G-F*, et al.* Electrochemical reduction of nitrate to ammonia via direct eight-electron transfer using a copper–molecular solid catalyst. *Nature Energy* **5**, 605-613 (2020).

6. Wang Y*, et al.* Enhanced Nitrate-to-Ammonia Activity on Copper–Nickel Alloys via Tuning of Intermediate Adsorption. *Journal of the American Chemical Society* **142**, 5702-5708 (2020).

7. McEnaney JM*, et al.* Electrolyte Engineering for Efficient Electrochemical Nitrate Reduction to Ammonia on a Titanium Electrode. *ACS Sustainable Chemistry & Engineering* **8**, 2672-2681 (2020).

8. Wang Y, Zhou W, Jia R, Yu Y, Zhang B. Unveiling the Activity Origin of a Copper-based Electrocatalyst for Selective Nitrate Reduction to Ammonia. *Angewandte Chemie International Edition* **59**, 5350-5354 (2020).

9. Li J*, et al.* Atomically dispersed Fe atoms anchored on S and N–codoped carbon for efficient electrochemical denitrification. *Proceedings of the National Academy of Sciences* **118**, e2105628118 (2021).

10. Wu Z-Y*, et al.* Electrochemical ammonia synthesis via nitrate reduction on Fe single atom catalyst. *Nature Communications* **12**, 2870 (2021).

11. Li P, Jin Z, Fang Z, Yu G. A single-site iron catalyst with preoccupied active centers that achieves selective ammonia electrosynthesis from nitrate. *Energy & Environmental Science* **14**, 3522-3531 (2021).

12. Lim J*, et al.* Structure Sensitivity of Pd Facets for Enhanced Electrochemical Nitrate Reduction to Ammonia. *ACS Catalysis* **11**, 7568-7577 (2021).

13. Li J*, et al.* Efficient Ammonia Electrosynthesis from Nitrate on Strained Ruthenium Nanoclusters. *Journal of the American Chemical Society* **142**, 7036-7046 (2020).

14. Chauhan R, Srivastava VC. Electrochemical denitrification of highly contaminated actual nitrate wastewater by Ti/RuO2 anode and iron cathode. *Chemical Engineering Journal* **386**, 122065 (2020).

15. Gao J*, et al.* Electrochemically Selective Ammonia Extraction from Nitrate by Coupling Electron- and Phase-Transfer Reactions at a Three-Phase Interface. *Environmental Science & Technology* **55**, 10684-10694 (2021).

16. Liu H*, et al.* Electrocatalytic Nitrate Reduction on Oxide-Derived Silver with Tunable Selectivity to Nitrite and Ammonia. *ACS Catalysis* **11**, 8431-8442 (2021).

17. ILLINOIS INTEGRATED WATER QUALITY REPORT AND SECTION 303(d) LIST, Clean Water Act Sections 303(d), 305(b) and 314, Volume II: Groundwater, Illinois Environmental Protection Agency Bureau of Water. (2016).

18. DECLINING GROUNDWATER QUALITY IN THE EASTERN SNAKE PLAIN AQUIFER - Causes, Trends, and Public Health Effects, Idaho Conservation League. (2019).

19. King KW, Williams MR, Fausey NR. Effect of crop type and season on nutrient leaching to tile drainage under a corn–soybean rotation. *Journal of Soil and Water Conservation* **71**, 56 (2016).
